# Phase I Trial of an Alhydrogel Adjuvanted Hepatitis B Core Virus-Like Particle Containing Epitopes of *Plasmodium falciparum* Circumsporozoite Protein

**DOI:** 10.1371/journal.pone.0001556

**Published:** 2008-02-06

**Authors:** Aric L. Gregson, Giane Oliveira, Caroline Othoro, J. Mauricio Calvo-Calle, George B. Thorton, Elizabeth Nardin, Robert Edelman

**Affiliations:** 1 Department of Medicine and Center for Vaccine Development, University of Maryland School of Medicine, Baltimore, Maryland, United States of America; 2 Department of Medical and Molecular Parasitology, New York University School of Medicine, New York, New York, United States of America; 3 Apovia, Inc., San Diego, California, United States of America; Mahidol University, Thailand

## Abstract

The objectives of this non-randomized, non-blinded, dose-escalating Phase I clinical trial were to assess the safety, reactogenicity and immunogenicity of ICC-1132 formulated with Alhydrogel (aluminum hydroxide) in 51 healthy, malaria-naive adults aged 18 to 45 years. ICC-1132 (Malariavax) is a recombinant, virus-like particle malaria vaccine comprised of hepatitis core antigen engineered to express the central repeat regions from *Plasmodium falciparum* circumsporozoite protein containing an immunodominant B [(NANP)_3_] epitope, an HLA-restricted CD4 (NANPNVDPNANP) epitope and a universal T cell epitope (T*) (amino acids 326—345, NF54 isolate). We assessed an Alhydrogel (aluminum hydroxide)-adjuvanted vaccine formulation at three ICC-1132 dose levels, each injected intramuscularly (1.0 mL) on study days 0, 56 and 168. A saline vaccine formulation was found to be unstable after prolonged storage and this formulation was subsequently removed from the study. Thirty-two volunteers were followed for one year. Local and systemic adverse clinical events were measured and immune responses to *P. falciparum* and hepatitis B virus core antigens were determined utilizing the following assays: IgG and IgM ELISA, indirect immunofluorescence against *P*. *falciparum* sporozoites, circumsporozoite precipitin (CSP) and transgenic sporozoite neutralization assays. Cellular responses were measured by proliferation and IL-2 assays. Local and systemic reactions were similarly mild and well tolerated between dose cohorts. Depending on the ICC-1132 vaccine concentration, 95 to 100% of volunteers developed antibody responses to the ICC-1132 immunogen and HBc after two injections; however, only 29—75% and 29—63% of volunteers, respectively, developed malaria-specific responses measured by the malaria repeat synthetic peptide ELISA and IFA; 2 of 8 volunteers had positive reactions in the CSP assay. Maximal transgenic sporozoite neutralization assay inhibition was 54%. Forty-seven to seventy-five percent demonstrated T cell proliferation in response to ICC-1132 or to recombinant circumsporozoite protein (rCS) NF-54 isolate. This candidate malaria vaccine was well tolerated, but the vaccine formulation was poorly immunogenic. The vaccine may benefit from a more powerful adjuvant to improve immunogenicity.

**Trial Registration:**

ClinicalTrials.gov NCT00587249

## Introduction

An effective vaccine is needed to prevent or attenuate disease from *Plasmodium falciparum* malaria, the most important cause of malaria morbidity and mortality throughout the world [1]. Protection from *P. falciparum* malaria infection and challenge was first demonstrated following immunization of humans with irradiation-attenuated *P. falciparum* sporozoites [2]. High levels of antibody directed against repeat regions of the circumsporozoite protein (CS) and high levels of interferon (IFN)-γ production by CD4^+^ and CD8^+^ cells against epitopes of CS, is associated with protection of humans and primates from *P. falciparum* malaria [3]–[7]. These findings suggest that a subunit vaccine which elicits robust humoral immunity directed against the extracellular sporozoite and robust cellular immunity with which to eliminate infected hepatocytes, could prevent patent blood-stage infection, the stage of the infection responsible for clinical illness.

Virus-like particles have been used recently as highly immunogenic delivery platforms for a variety of vaccines [8]–[13]. The virus-like particle malaria vaccine RTS,S is composed of hepatitis B virus surface antigen which contains the CS repeat and C terminus region (amino acids 207—395) of the *P. falciparum* NF54 isolate (3D7 clone). In combination with potent proprietary adjuvants, this vaccine has protective efficacy against malarial disease and severe malaria [14]–[17]. Complete protection was obtained in 40% of immunized malaria naive volunteers undergoing sporozoite challenge [15], [18] and 30% protection against the first clinical episode of malaria for 18 months in children living in malaria endemic areas [16], [17]. More robust vaccines capable of greater and longer duration of protection are sought through more effective vaccine delivery platforms. The hepatitis B virus core protein (HBc) has been demonstrated to be an effective malaria vaccine platform in animals where high levels of anti-CS repeat antibodies protected animals from malaria challenge [19], [20].

Circumsporozoite protein is comprised of a central portion of amino acid repeats (NANP) representing dominant T cell-dependent B cell epitopes [21], [22]. T cell epitopes have been identified in the CS molecule, which are HLA-restricted CD8^+^ and CD4^+^ T cell epitopes, as well as universal CD4^+ ^T cell epitopes [6], [23]–[25]. The present vaccine, ICC-1132, was conceived in an effort to boost antibody levels and generate a robust cellular immune response. It is comprised of central repeat regions of CS containing (1) both immunodominant B (NANP)_3_ and HLA-restricted CD4^+^ T cell (NANPNVDP) epitopes identified from irradiated sporozoite immunization studies [5], [21], (2) a universal T cell epitope (T*) from the carboxyl terminus of CS (amino acids 326—345 NF54 isolate) containing CD4^+^ T cell epitopes, which bind to a wide range of HLA types [24], [26], [27], and (3) at least one CD8^+^ T cell epitope [28]. These CS epitopes are inserted into a HBc backbone which spontaneously aggregates to form virus-like particles. This vaccine was found to be highly immunogenic in rodent and non-human primates [29].

After initiation of this present study in the USA, the ICC-1132 vaccine was tested in other, more limited studies in Europe, the results of which have been published [30]–[32]. A study in Cardiff, Wales used the same protocol as the present study, but included only the 20 and 50 mcg vaccine doses with the same Alhydrogel adjuvant [30]. A limited analysis of cellular immune responses was carried out using IFN-γ ELISpot. Immunogenicity of a single injection of ICC-1132 formulated with Seppic ISA 720 adjuvant, instead of Alhydrogel, was assessed in a Phase 1 dose response study [32] and in a Phase 1/2 trial of a single dose of 50 mcg/ISA 720 [31]. The present study was initially designed to be a phase 1/2 study of the three ICC-1132 concentrations in either saline or adsorbed to Alhydrogel, followed by a malaria challenge trial. Because the malaria challenge was not performed, this report summarizes the results of the phase 1 trial of ICC-1132 in the United States.

## Methods

The protocol for this trial and supporting CONSORT checklist are available as supporting information; see Checklist S1 and Protocol S1.

### Participants

The trial took place on the campuses of the University of Maryland at Baltimore and at College Park. The study methods and rationale, along with the study consent was explained to potential healthy, adult participants aged 18—45. A written exam was administered to potential participants to assess their understanding of the study procedures, rationale and expected outcomes. Consented participants were screened by medical history, physical examination and laboratory analysis of hematologic and serologic markers. Exclusion criteria included medical history or serologic indications of malaria, HIV, HBsAg or any significant cardiovascular, hepatic or renal function abnormalities.

### Interventions

#### Vaccine Construct

**Figure 1:**
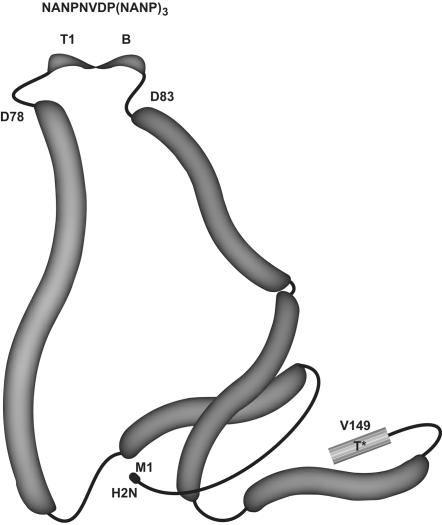
The T1(B)_3_ repeat epitopes, NANPDVDP(NANP)_3_, are inserted between amino acid residues 78 and 82 of the hepatitis B core protein, forming the tip of the core antigen spikes. The T* epitope, EYLNKIQNSLSTEWSPCSVT, is inserted starting at amino acid V149. The amino terminus is noted as H2N and the starting amino acid is labelled as M1. Adapted from Bottcher, *et al*.

The vaccine has been described [29]. In summary, recombinant ICC-1132 is comprised of the assembly domain of the HBc gene (amino acid residues 1—149) with the CS universal T cell epitope (T*) (CS amino acid residues 326—345) fused to the HBc C-terminus following Val_149_ [33] (Figure 1). The B and T cell CS repeat epitopes, T1 (NANPNVDPNANP) and B [(NANP)_3_], are inserted into the HBc immunodominant loop between amino acid residues Asp_78_ and Pro_79_. Recombinant HBc dimers self-assemble into an icosahedral virus-like particle of approximately 30 nm in diameter composed of 180—240 individual copies of the recombinant protein [29], [33]. Based on HBc structure, the CS repeats contained in ICC-1132, inserted between amino acid residues 78 and 82 of HBc, are believed to be localized to the tip of surface spikes on the particle formed by dimerization of HBc monomers [33], [34]. The T* epitope replaces the HBc protamine-like domain (HBc amino acid residues 150—183) and is therefore most likely oriented to the inner surface of the core particle, which is believed to be similar to its orientation in the native circumsporozoite protein.

ICC-1132 was adsorbed to aluminum hydroxide (Alhydrogel; Superfos, Frederikssund, Denmark) with >95% adsorption as determined by measurement of residual unbound protein. Each 1 mL of vaccine contained 1 mg of aluminum as Al(OH)_3_. The saline formulation contained the same concentration of ICC-1132, but did not contain Alhydrogel. The vaccine was produced, purified and formulated by Apovia, Inc.

#### Study Design

The study was originally designed as a double-masked, dose-escalating trial comparing the three dose levels (10, 20 and 50 mcg) of ICC-1132 in saline to the same three dose levels of ICC-1132 adsorbed to Alhydrogel. In January 2003 the trial was delayed because the saline formulation of the vaccine was found to be unstable after six to nine months' storage at 4°C. The saline formulation was subsequently removed from the study, leaving the stable alum preparation to be tested. The study was redesigned to assess the 20 and 50 mcg doses of the Alhydrogel vaccine in a non-masked, dose escalating fashion. The study was conducted according to GCP guidelines. Ethical approval for the study was obtained from the Institutional Review Boards of the University of Maryland, Baltimore and University of Maryland, College Park and the New York University School of Medicine.

### Objectives

The primary objectives were (1) to compare the safety and reactogenicity of ICC-1132 in saline and with the Alhydrogel formulation in healthy, malaria-naive human adults, and (2) to assess the immunogenicity of the two ICC-1132 vaccine formulations. A secondary objective was to vaccinate a sufficient number of volunteers who would agree to participate in a concurrent malaria challenge trial. The immunogenicity data for the saline formulation is abbreviated for reasons described. The malaria challenge trial was cancelled because of lack of sufficient immunogenicity, predefined as a dose cohort median IFA of ≥1500.

### Outcomes

#### Safety and Reactogenicity

Local and systemic reactions were assessed for 30 minutes following each injection. Clinical assessments were carried out at 1, 2, 7, 14, 28 and 56 days after each injection, at day 84 after the second injection, and day 168 after the third injection. Telephone interviews were conducted at 4 days after each injection and volunteers maintained a daily diary to collect adverse events and twice daily body temperature recordings in the seven days immediately after each injection.

#### Local Reactions

Tenderness, pain, erythema, induration and pruritus, at the site of injection, were graded.

#### Systemic Reactions

Solicited systemic variables evaluated included fever, chills, malaise, headache, photophobia, anorexia, nausea, vomiting, abdominal pain, myalgia, arthralgia and rash. Non-solicited adverse event reported by a volunteer was recorded and an assessment of causality was performed by the investigators.

#### Local and Systemic Events Grading

Local and systemic adverse events were graded according to the following schema: Grade 1 mild, no change in activity and/or no medication necessary; Grade 2 moderate, requires change in activity and/or medication; Grade 3 severe, bed rest required/inability to perform normal activities and/or medical intervention other than medication alone (such as an outpatient visit in emergency department or clinic, excluding hospitalization).

#### Clinical Chemistry

Urinalysis, hematological and biochemical safety analysis was carried out prior to vaccine injections, 2, 14, 28 days after all injections, 84 days after the second injection, and 56 and 168 days after the third injection.

#### Immunogenicity – Serologic Assays

Serum samples for serological assays and peripheral blood mononuclear cells (PBMC) for cellular immune assays were obtained at the time of each immunization, 14 and 28 days after each injection, 84 days after the second injection, and 56 and 168 days after the third injection. Methods used for ELISA, IFA and CSP reactions are identical to those previously published [30]. Significant values were taken to be a ≥4-fold increase from baseline titers. Antibody reactivity with viable sporozoites was assessed by circumsporozoite precipitin (CSP) reaction in which antibody mediated cross linking of surface CS protein results in formation of a terminal precipitin reaction on sporozoites detectable by phase microscopy [35]. The presence of neutralizing antibodies in immune sera was determined using a Transgenic Sporozoite Neutralization Assay (TSNA) based on transgenic rodent *Plasmodium berghei* sporozoites expressing *P. falciparum* CS repeats [36], [37]. In the TSNA, 2x10^4^ transgenic sporozoites were incubated in serum (1:5 dilution) from each volunteer or with controls of medium only or 25 mcg/mL of either mAb 3D11, specific for *P. berghei* repeats, or mAb 2A10, specific for *P. falciparum* repeats. Sporozoites were incubated with serum, mAb or medium for 40 minutes on ice prior to their being added to human hepatoma cell (HepG2) cultures. The cultures were incubated for 72 hours followed by extraction of total RNA from the cells for determination of intracellular parasites by RT-PCR using primers specific for parasite 18S ribosomal RNA. All TSNA were performed in duplicate. Inhibition of >85% is considered positive.

#### Immunogenicity – Cellular Assays

The PBMC were Ficoll purified from blood collected in citrate buffer Vacutainer (BD Biosciences, San Diego, CA). Short term TCL were used in the assays by expanding PBMC (2x10^6^/mL) with a single *in vitro* stimulation with rCS (10 mcg/mL) with recombinant human interleukin-2 (IL-2) added on day five [32], [38]. In the proliferation assay, the short term TCL were incubated in triplicate wells with various concentrations of rCS or HBc proteins or peptides representing the CS repeats (T1B)_4_ or the universal T cell epitope (T*). Culture wells tritiated with ^3^H-Tdr on day five were incubated overnight and harvested. The results are expressed as delta (δ) cpm (cpm in cultures stimulated with antigen – cpm in cultures without antigen) or stimulation index (SI) (cpm in antigen stimulated cultures/cpm induced by supernatants from medium only cultures). IL-2 was measured in a bioassay using 24 hour cell culture supernatants incubated with an IL-2 dependent cell line [32], [38] and the results expressed as δ cpm or SI. Significant responses were taken as δ cpm>mean+2 standard deviations of responses obtained with pre-immune cells from the volunteers.

### Sample size

In this phase 1 descriptive study focusing on safety and immunogenicity, sample sizes were derived from logistic considerations, rather than by power analyses. The larger number of volunteers in the 50 mcg cohort was designed to yield sufficient interested volunteers to enable a malaria challenge trial after the third vaccination.

### Randomization—Sequence generation

Using SAS, a randomization list was prepared in advance of vaccination activities with randomized block of *N* = 2 (one subject receiving saline and the other Alhydrogel formulated vaccine) for the August 2002 cohorts.

### Randomization—Allocation concealment

Assignments to vaccine groups from this randomization were placed in individual sealed envelopes and were provided to the immunization team. Randomization assignment was masked from investigators assessing post-injection adverse events.

### Randomization—Implementation

The original study statistician generated the allocation sequence. Subjects in the 10 mcg cohort and the first three subjects in the 20 mcg cohort were randomized by computer to receive ICC-1132 in saline or ICC-1132 adsorbed to Alhydrogel. All 32 subsequent subjects received ICC-1132 adsorbed to Alhydrogel, and were allocation to either the 20 mcg or 50 mcg cohorts on a first come, first serve basis, such that the first eight available subjects were assigned the 20 mcg cohort, while the last 24 subjects were assigned the 50 mcg cohort.

### Blinding

The participants, vaccine administrators and clinical staff providing safety follow-up were blinded to which formulation of vaccine subjects had received prior to removal of the saline formulated vaccine from the study. Neither the subjects nor investigators were blinded once the saline formulation was removed from the trial, because only one formulation was used and because the dose-escalation design required that the safety of the 20 mcg dose be evaluated prior to administration of the 50 mcg dose.

### Statistical methods

Quantitative data were assessed for normal distribution and log-transformation was performed where appropriate. Differences among proportions were compared using Fisher's exact tests, differences between medians were compared using the Wilcoxon Rank Sum test and Spearman's Rank Correlation test was used to assess association strengths. All tests were two sided. A *P*≤0.05 was taken to be statistically significant. R v2.2.0 [39] was used for statistical analysis.

## Results

### Participant Flow and Recruitment

**Figure 2:**
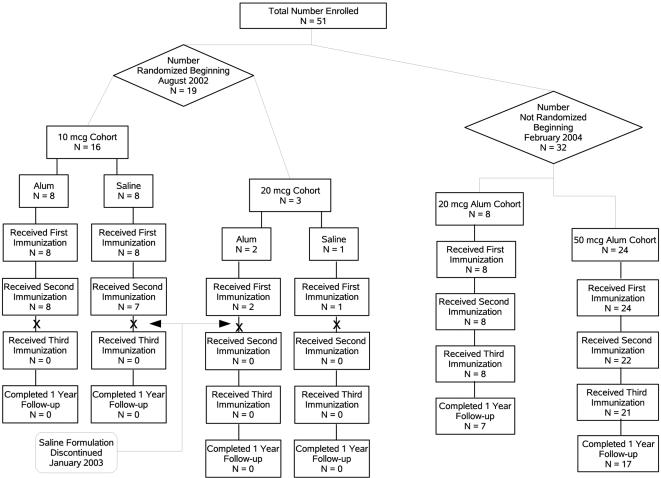
Consort Flow Diagram. A total of 51 volunteers received at least one injection and 29 volunteers completed the study to receive all three injections of ICC-1132 adjuvanted to Alhydrogel.

A total of 51 volunteers received at least one injection and 29 volunteers completed the study to receive all three injections of ICC-1132 adjuvanted to Alhydrogel (Figure 2).

### Baseline demographic data

The distribution of volunteers by gender and ethnicity was similar statistically amongst all dose and formulation groups (*P* = 0.76). The median age of all trial participants was 25 years, with a range of 18—45 years. There was a statistically significant difference in participant age between the Baltimore (median age 26.5 [24 to 31]) and College Park (median age 21 [21 to 23]) cohorts (*P* = 0.008).

### Safety

The vaccine was well tolerated at the dose levels examined in this study. No severe (grade 3) adverse events occurred, nor any clinically significant laboratory abnormalities attributable to vaccination. One serious adverse event occurred, unrelated to vaccination, in a volunteer who became pregnant within three months of receiving the third 20 mcg injection. She delivered a healthy infant at 40 weeks. No significant differences in reactogenicity were noted between dose cohorts, between the first, second and third vaccination, nor between Alhydrogel and saline vaccine groups.

#### Local Reactogenicity

Of 125 total vaccinations administered to 51 volunteers, local adverse events were principally limited to mild tenderness or pain at the injection site in 46 volunteers. In addition, four volunteers had local pruritus and one volunteer each had an episode of induration and erythema (Table 1). Most reactions were mild and lasted 1—3 days. There were no severe reactions.

**Table 1:** Local Adverse Events by Episode in 125 Vaccinations

	Local				
	Tenderness	Pain	Pruritus	Induration	Erythema
Grade 1*	68	53	4	1	1
Grade 2†	1	9	0	0	0
Grade 3‡	0	0	0	0	0
Total	69	62	4	1	1

* Grade 1: Mild; no change in activity and/or no medication necessary

† Grade 2: Moderate; requires change in activity and/or medication

‡ Grade 3: Severe; bed rest required and/or medical intervention other than medication alone (such as an outpatient visit in emergency department or clinic, excluding hospitalization)

#### Systemic Reactogenicity

Headache and myalgia were the most common manifestations of systemic reactogenicity, followed by other elicited symptoms (Table 2). Symptoms were generally mild and persisted 1—5 days. There were no severe or serious reactions. Because no placebo group was included it is difficult to ascribe causality to the vaccine. No delayed reactogenicity was reported by the 28 volunteers who were contacted on day 336 follow-up (168 days after the third vaccination).

**Table 2:** Systemic Adverse Events by Episode in 125 Vaccinations

	Systemic					
	HA*	Myalgia	Subjective Fever†	Nausea	Arthralgia	Anorexia
Grade 1	14	12	4	3	4	4
Grade 2	6	3	1	3	0	0
Grade 3	0	0	0	0	0	0
Total	20	15	5	6	4	4

* HA = headache

† One episode of temperature to 37.8 C

### Immunogenicity

#### Humoral Immune Responses

Depending upon the vaccine dose, 95—100% of volunteers developed specific responses to the ICC-1132 vaccine immunogen (Table 3). However, only 50—75% of the volunteers developed (T1B)_4_ malaria specific responses, while 29—63% developed IFA malaria specific responses. There was no significant boosting of malaria specific humoral immune responses [(T1B)_4_ and IFA] after the third injection of ICC-1132 (Table 3). Less than a four-fold boosting of anti-HBc titers occurred after the third injection in both the 20 and 50 mcg cohorts. This increase in anti-HBc titer was statistically significant in the 50 mcg cohort (*P* = 0.02).

**Table 3:** GMT of All Vaccinees (Percent Responders) Against Immunogen (ICC-1132), Hepatitis Core (HBc), Malaria Repeat Antigen ((T1B)_4_) and Whole Sporozoite (IFA)

Antigen	ICC-1132*			HBc*			(T1B)_4_ *			IFA†		
Post Dose	1	2	3	1	2	3	1	2	3	1	2	3
10 µg	320	16255‡,§	NA	63	4561¶,||	NA	67	269**	NA	NA	226	NA
	(88)	(100)	-	(33)	(100)	-	(25)	(75)	-	-	(63)	-
20 µg	123	905‡	3121	73	905¶	2319	<80	106	101	NA	80	66
	(50)	(100)	(100)	(50)	(100)	(100)	(13)	(50)	(43)	-	(25)	(29)
50 µg	88	1004§	2661	49	577||	2463	45	63**	138	NA	57	118
	(48)	(95)	(94)	(17)	(90)	(94)	(8)	(38)	(53)	-	(25)	(53)

* ELISA

† Indirect immunofluorescence assay

‡ *P = *0.008, between ICC-1132 10 and 20 µg cohorts post dose 2

§ *P = *0.001, between ICC-1132 10 and 50 µg cohorts post dose 2

¶ *P = *0.03, between HBc 10 and 20 µg cohorts post dose 2

|| *P = *0.007, between HBc 10 and 50 µg cohorts post dose 2

** *P = *0.019, between (T1B)_4_ 10 and 50 µg cohorts post dose 2

Differences between 10, 20 and 50 µg cohort percent responders not statistically significant

Vaccination on days 0, 56 and 168

NA = Not available

The anti-ICC-1132 and anti-HBc GMTs were significantly higher in the 10 mcg cohort compared to the 20 and 50 mcg cohorts after the second injection, although the percent responders to the two antigens (90—100%) were nearly identical in the three cohorts (Table 3). The 10 mcg cohort was not vaccinated a third time. The anti-ICC-1132 and anti-HBc GMTs and percent responders in the 20 and 50 mcg cohorts after each of three injections were similar.

Malaria specific anti-(T1B)_4_ response was significantly higher in the 10 mcg cohort compared to the 50 mcg cohort after the second injection (*P* = 0.019) (Table 3). Although the IFA GMT and percent responders were higher in the 10 mcg as compared to the 20 and 50 mcg cohorts, the differences were not statistically significant. Overall, the malaria specific responses were low in comparison to immunogen specific responses.

Anti-ICC-1132, anti-HBc and anti-(T1B)_4_ IgG1 and IgG3 subtypes, typical of T_H_1-type immune responses, developed preferentially over IgG2 and IgG4 subtypes (*P* = 3.33^−5^) without affect of the adjuvant (*P* = 0.21) (titer expressed in O.D.; data not shown).

**Figure 3:**
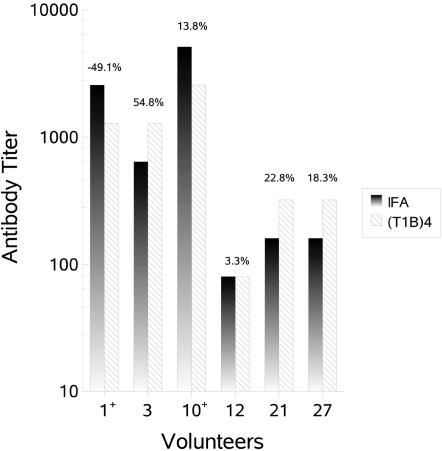
Comparison of Antibody Titer and Sporozoite Neutralizing Activity for Select Volunteers. As measured by IFA against whole sporozoite (gradient bars), (T1B)4 ELISA (hatched bars) and transgenic sporozoite neutralization assay (TSNA) (percentages above bars). For the TSNA, 94.5% inhibition was obtained using a positive control monoclonal antibody specific for *P. falciparum* CS repeats and −5% for negative control MAB specific for *P. berghei* CS repeats. Volunteers with positive CSP assays, volunteers 1 and 10, are indicated with a plus sign (+) in the *x*-axis.

Two (volunteers 1 and 10) of the seven volunteers in the 10 mcg alum cohort (those with the most robust malaria specific humoral responses) developed positive CSP reactions, which demonstrates the presence of specific antibodies capable of cross-linking surface CS protein on the viable sporozoite. Inhibition in the TSNA using *PfPb* sporozoite invasion of HepG2 cells ranged from a 54% inhibition (volunteer 3) to a 49% increase (volunteer 1) in 18S rRNA copies at 28 days post-vaccination two (10 mcg cohort) or 28 days post-vaccination three (20 mcg cohort) (Figure 3). TSNA data for the 50 mcg cohort is not available. There was a correlation between IFA titer and CSP reactions, though only two volunteers in the 10 mcg cohort had positive CSP reactions (ρ =  0.70, *P* = 0.003). The two highest responders by IFA and those with the positive CSP reactions, volunteers 1 and 10, demonstrated little to no inhibition by the TSNA (Figure 3).

#### Cellular Immune Responses

Depending on vaccine dose, 65—75% of the volunteers' demonstrated T cell proliferation in response to ICC-1132 and 47—75% had proliferation to rCS. The magnitude of responses to these two antigens was similar (Table 4). Seven of 28 volunteers gave a T cell response to the universal T cell epitope (T*)_4_, as measured by IL-2 bioassays (Table 5). Only one volunteer had a consistent response to the restricted CS repeat (T1B)_4_. Fifty-six days after the third injection of the ICC-1132 vaccine no volunteer had significant cellular immune responses in either the proliferation or the IL-2 bioassay (data not shown). There was good correlation between the proliferation assays and IL-2 bioassays (ρ = 0.5—0.9, for individual cohorts), though the *P* values for the 10 mcg cohort correlations were >0.05.

**Table 4:** TCL Proliferation Median δCPM (Percent Responders) All Vaccinees Days 84 and 196

Cohort	Day 0		Day 84		Day 196	
	ICC-1132	rCS	ICC-1132	rCS	ICC-1132	rCS
10 µg*	3033	4459	21744‡	23496‡	NA	NA
	-	-	(75)	(75)	-	-
20 µg†	587	3484	15903	4736	10451	13772
	-	-	(67)	(75)	(57)	(29)
50 µg	310	462	3636§	11948¶	10035||	11063||
	-	-	(13)	(29)	(65)	(47)

* Data from 4 volunteers

† Data from 7 volunteers

‡ Responses 14 days after the second dose of ICC-1132 (Day 70)

§ Data from 8 volunteers

¶ Data from 14 volunteers

|| Data from 17 volunteers

** Differences between 10, 20 and 50 µg cohorts *δ*CPM & percent responders not statistically significant

Vaccination on days 0, 56 and 168

NA = Not available

**Table 5:** Fine Specificity of Malaria Specific Responses: Median IL-2 CPM All Vaccinees (Number of Responders/Number of Total, as measured by δCPM)

Cohort	Antigen		
	rCS NF-54	(T*)_4_ *	(T1B)_4_
10 µg†	12114 (0/4)	47 (1/4)	52 (0/4)
20 µg‡	1515 (1/7)	268 (2/7)	242 (1/7)
50 µg‡	2831 (7/17)	147 (4/17)	55 (0/17)
Total Percent Responders	29 (8/28)	25 (7/28)	4 (1/28)

* T universal epitope

† Responses 14 days after the second dose of ICC 1132 (Day 70)

‡ Responses 28 days after the third dose of ICC 1132 (Day 196)

Differences between cohorts not statistically significant

## Discussion

### Interpretation

This *P. falciparum* CS-based malaria vaccine, a novel virus-like particle, was safe and well tolerated. It elicited robust antibody responses to the immunogen (ICC-1132) and to HBc, but the malaria specific antibody responses were relatively weak. For example, 95—100% of all volunteers developed specific responses to the ICC-1132 vaccine immunogen and to HBc, but only 50—75% developed anti-CS repeat responses and 29—63% developed IFA sporozoite responses (Table 3). The geometric mean titers by IFA and rCS ELISA were suboptimal, arbitrarily defining an IFA titer of 1500 as minimal adequate response for malaria challenge, and only weak boosting was seen after the third vaccination. In cellular responses, 8 of 28 (29%) and 7 of 28 (25%) responded to rCS and the universal T cell epitope (T*)_4_ malarial antigens respectively (Table 5). Because only a limited number of class II genotypes can function as restriction elements for the CS repeat epitope, it was not unexpected to find only 1 of 28 persons consistently respond to the HLA-restricted (T1B)_4_ antigen (Table 5). Although the cellular proliferation response to the ICC-1132 and rCS antigens were similar, this candidate malaria vaccine did not achieve our predefined criteria, a median IFA response of >1:1500, to justify a malaria challenge.

### Overall evidence

#### Intercohort Differences

Our most intriguing finding is the difference between the 10 and 50 mcg cohorts in terms of humoral immunogenicity of the vaccine. For all antigens evaluated, the 10 mcg cohort response was superior to that of the 50 mcg after the second injection. Intercohort differences may have been an effect of the manner in which antigen bound to the adjuvant in the lower versus higher dose vaccines. Although unusual, it is not without precedent that a lower immunogen dose would lead to a more robust humoral response [40]–[42]. The vaccine lots differed between the 10 mcg and the 20 and 50 mcg cohorts because of the trial delay. The 10 mcg vaccine lot in our study was the same as was used in the Cardiff study of 20 and 50 mcg cohorts [30] so it is helpful to compare these results with our own. There remains a statistically significant difference between our 10 mcg and the Cardiff 20 and 50 mcg cohorts when comparing ICC-1132 GMT at day 84 (GMT 989 and 1522 in the Cardiff 20 and 50 mcg cohorts respectively) (*P*<0.035 in all cases), but there were no statistically significant differences in the percent responders.

#### Hepatitis B Core Effects

Volunteers with preexisting anti-HBc titers (two volunteers in the 10 alum and one volunteer in the 50 mcg cohort) demonstrated more robust humoral responses to immunogen and HBc, with titers consistently higher than their respective cohorts' GMT. There was not a noticeably increased response in these individuals against malaria-specific antigens. This suggests that, in these previously anti-HBc positive individuals, the more robust response against the immunogen was specific for HBc epitopes. Indeed, the higher antibody titers seen against both the immunogen and HBc in all the volunteers could imply that the targeted epitopes in the immunogen are shared HBc epitopes, rather than malaria epitopes. The proliferation data shows that the cellular immune response is targeting malaria-specific responses, as the median δCPM against rCS and ICC-1132 are similar at day 196 (Table 4). As has been demonstrated in other studies, the cellular immune response against *P. falciparum* sporozoites is likely a critical component of protection [7], [43], [44]. Analysis of cytokine and IFN ELISpot will be reported in a separate paper (C. Othoro, manuscript in preparation).

#### T_H_1 Type Response

Volunteers developed T_H_1-type antibody, with anti-CS IgG1 and IgG3 subtypes developing preferentially over IgG2 and IgG4, as found in previous study of this vaccine [30]. This effect was independent of the adjuvant, as similar predominance of IgG1 and IgG3 was note din the saline group (data not shown), suggesting a T_H_1 inducing property of the core particle [45]. The practical effect of a preferential T_H_1-type antibody response would be production of gamma interferon, a known inhibitor of hepatic stage intracellular parasites [43], [46], [47].

#### Functional Assays

The good correlation between (T1B)_4_ ELISA and IFA titers suggests that anti-repeat antibodies elicited by the ICC-1132 vaccine recognize native CS on the sporozoite (Figure 3). Two individuals with the highest IFA titers gave a positive CSP reaction [30]. There was no correlation between either IFA or CSP reaction and level of inhibition in the TSNA using *PfPb* sporozoite invasion of HepG2 cells (Figure 3). It is possible that our antibody titers and/or avidity were not sufficient to lead to inhibition in the TSNA. In a previous study, antibodies developed against a (T1B)_4_ MAP [27] vaccine demonstrated high level inhibition of invasion of HepG2 cells in the TSNA [37], however challenge studies were not carried out in that study and correlation with protection *in vivo* remains unknown. In malaria blood stage vaccines, functional assays based on transgenic parasites expressing falciparum blood stage epitopes were more predictive of protection, presumably because the ELISA does not measure functional antibodies [48], [49]. As yet there remains no accurate correlate of protection for malaria sporozoite challenge [15], [18], [50], [51]. Previous studies have attempted to correlate protection and inhibition of invasion of *P. falciparum* sporozoites into hepatoma cells (ISI) assay [52]–[56]. Still others have suggested the use of IFN-γ ELISpot to measure T cell responses or opsonizing anti-CS antibodies as correlates of protection [7], [57]. Proper correlates of protection may well vary depending upon the antigen/adjuvant combination tested.

#### Comparison to Other Studies of Same Immunogen

After initiation of this present study in the USA, the vaccine was tested in other, more limited studies in Europe, the results of which have been published [30]–[32]. One study assessed immunogenicity of a single injection of 5, 20 or 50 mcg of ICC-1132 formulated with the more potent adjuvant, Seppic ISA 720, instead of Alhydrogel [32]. A single dose of this 50 mcg ICC-1132/ISA 720 elicited maximal GMT anti-repeat ELISA titers of 1050 (100% seroconversion), higher than in the current study with maximal anti-(T1B)_4_ GMT of 269 (75% seroconversion). In contrast, our alum formulation elicited peak anti-HBc responses that were more robust, exceeding 16000 GMT following booster immunization, as compared to peak anti-HBc GMT of 830 elicited by a single immunization with the ISA 720 formulation. In a second study of the ISA 720 formulation, 9 of 11 volunteers receiving a single 50 mcg injection of ICC-1132/ISA 720 developed positive anti-repeat antibody responses (GMT 370) but minimal T cell IFN ELISpot responses [31]. Following challenge with *P. falciparum* sporozoite, there was no appreciable effect of vaccination on malaria infection, indicating that multiple immunization and/or higher antibody titers or more robust cellular immune responses are required for protection.

In summary, the candidate vaccine, ICC-1132, was safe and well-tolerated at all dose levels examined in this trial. ICC-1132 was poorly immunogenic when adjuvanted with alum. The immunogenicity of the candidate vaccine may be improved through combination with a more potent adjuvant.

## Supporting Information

Checklist S1 CONSORT Checklist
(0.10 MB PDF)

Protocol S1 Final Trial Protocol
(0.78 MB PDF)

## References

[1] Breman J (2001). The ears of the hippopotamus: Manifestations, determinants, and estimates of the malaria burden.. Am J Trop Med Hyg.

[2] Clyde D, V M, Miller R, Hornick R (1973). Specificity of protection of man immunized against sporozoite-induced falciparum malaria.. Am J Med Sci.

[3] Nardin EH, Nussenzweig V, Nussenzweig RS, Collins WE, Harinasuta KT (1982). Circumsporozoite proteins of human malaria parasites plasmodium falciparum and plasmodium vivax.. J Exp Med.

[4] Herrington DA, Clyde DF, Losonsky G, Cortesia M, Murphy JR (1987). Safety and immunogenicity in man of a synthetic peptide malaria vaccine against plasmodium falciparum sporozoites.. Nature.

[5] Nardin E, Herrington D, Davis J, Levine M, Stuber D (1989). Conserved repetitive epitope recognized by CD4+ clones from a malaria-immunized volunteer.. Science.

[6] Nardin E, Nussenzweig R (1993). T cell responses to pre-erythrocytic stages of malaria: Role in protection and vaccine development against pre-erythrocytic stages.. Annu Rev Immunol.

[7] Sun P, Schwenk R, White K, Stoute J, Cohen J (2003). Protective immunity induced with malaria vaccine, RTS,S, is linked to plasmodium falciparum circumsporozoite protein-specific CD4+ and CD8+ T cells producing IFN-gamma.. J Immunol.

[8] Koutsky LA, Ault KA, Wheeler CM, Brown DR, Barr E (2002). A controlled trial of a human papillomavirus type 16 vaccine.. N Engl J Med.

[9] Galarza JM, Latham T, Cupo A (2005). Virus-like particle vaccine conferred complete protection against a lethal influenza virus challenge.. Viral Immunol.

[10] Goldberg SM, Bartido SM, Gardner JP, Guevara-Patino JA, Montgomery SC (2005). Comparison of two cancer vaccines targeting tyrosinase: Plasmid DNA and recombinant alphavirus replicon particles.. Clin Cancer Res.

[11] Huang Y, Liang W, Wang Y, Zhou Z, Pan A (2005). Immunogenicity of the epitope of the foot-and-mouth disease virus fused with a hepatitis B core protein as expressed in transgenic tobacco.. Viral Immunol.

[12] Vuola JM, Keating S, Webster DP, Berthoud T, Dunachie S (2005). Differential immunogenicity of various heterologous prime-boost vaccine regimens using DNA and viral vectors in healthy volunteers.. J Immunol.

[13] Bellier B, Dalba C, Clerc B, Desjardins D, Drury R (2006). DNA vaccines encoding retrovirus-based virus-like particles induce efficient immune responses without adjuvant.. Vaccine.

[14] Bojang K, Milligan P, Pinder M, Vigneron L, Alloueche A (2001). Efficacy of RTS,S/AS02 malaria vaccine against plasmodium falciparum infection in semi-immune adult men in the gambia: A randomised trial.. Lancet.

[15] Kester KE, McKinney DA, Tornieporth N, Ockenhouse CF, Heppner DG (2001). Efficacy of recombinant circumsporozoite protein vaccine regimens against experimental plasmodium falciparum malaria.. J Infect Dis.

[16] Alonso P, Sacarlal J, Aponte J, Leach A, Macete E (2004). Efficacy of the RTS,S/AS02A vaccine against *plasmodium falciparum* infection and disease in young african children: Randomised controlled trial.. Lancet.

[17] Alonso PL, Sacarlal J, Aponte JJ, Leach A, Macete E (2005). Duration of protection with RTS,S/AS02A malaria vaccine in prevention of plasmodium falciparum disease in mozambican children: Single-blind extended follow-up of a randomised controlled trial.. Lancet.

[18] Stoute J, Slaoui M, Heppner D, Momin P, Kester K (1997). A preliminary evaluation of a recombinant circumsporozoite protein vaccine against plasmodium falciparum malaria. RTS,S malaria vaccine evaluation group.. N Engl J Med.

[19] Schodel F, Wirtz R, Peterson D, Hughes J, Warren R (1994). Immunity to malaria elicited by hybrid hepatitis B virus core particles carrying circumsporozoite protein epitopes.. J Exp Med.

[20] Schodel F, Peterson D, Milich DR, Charoenvit Y, Sadoff J (1997). Immunization with hybrid hepatitis B virus core particles carrying circumsporozoite antigen epitopes protects mice against plasmodium yoelii challenge.. Behring Inst Mitt.

[21] Zavala F, Cochrane AH, Nardin EH, Nussenzweig RS, Nussenzweig V (1983). Circumsporozoite proteins of malaria parasites contain a single immunodominant region with two or more identical epitopes.. J Exp Med.

[22] Molano A, Park SH, Chiu YH, Nosseir S, Bendelac A (2000). Cutting edge: The IgG response to the circumsporozoite protein is MHC class II-dependent and CD1d-independent: Exploring the role of GPIs in NK T cell activation and antimalarial responses.. J Immunol.

[23] Sinigaglia F, Guttinger M, Kilgus J, Doran DM, Matile H (1988). A malaria T-cell epitope recognized in association with most mouse and human MHC class II molecules.. Nature.

[24] Calvo-Calle J, Hammer J, Sinigaglia F, Clavijo P, Moya-Castro Z (1997). Binding of malaria T cell epitopes to DR and DQ molecules in vitro correlates with immunogenicity in vivo: Identification of a universal T cell epitope in the plasmodium falciparum circumsporozoite protein.. J Immunol.

[25] Doolan DL, Hoffman SL, Southwood S, Wentworth PA, Sidney J (1997). Degenerate cytotoxic T cell epitopes from P. falciparum restricted by multiple HLA-A and HLA-B supertype alleles.. Immunity.

[26] Moreno A, Clavijo P, Edelman R, Davis J, Sztein M (1993). CD4+ T cell clones obtained from plasmodium falciparum sporozoite-immunized volunteers recognize polymorphic sequences of the circumsporozoite protein.. J Immunol.

[27] Nardin EH, Oliveira GA, Calvo-Calle JM, Castro ZR, Nussenzweig RS (2000). Synthetic malaria peptide vaccine elicits high levels of antibodies in vaccinees of defined HLA genotypes.. J Infect Dis.

[28] Blum-Tirouvanziam U, Servis C, Habluetzel A, Valmori D, Men Y (1995). Localization of HLA-A2.1-restricted T cell epitopes in the circumsporozoite protein of plasmodium falciparum.. J Immunol.

[29] Birkett A, Lyons K, Schmidt A, Boyd D, Oliveira GA (2002). A modified hepatitis B virus core particle containing multiple epitopes of the plasmodium falciparum circumsporozoite protein provides a highly immunogenic malaria vaccine in preclinical analyses in rodent and primate hosts.. Infect Immun.

[30] Nardin E, Oliveira G, Calvo-Calle J, Wetzel K, Maier C (2004). Phase I testing of a malaria vaccine composed of hepatitis B virus core particles expressing plasmodium falciparum circumsporozoite epitopes.. Infect Immun.

[31] Walther M, Dunachie S, Keating S, Vuola J, Berthoud T (2005). Safety, immunogenicity and efficacy of a pre-erythrocytic malaria candidate vaccine, ICC-1132 formulated in seppic ISA 720.. Vaccine.

[32] Oliveira GA, Wetzel K, Calvo-Calle JM, Nussenzweig R, Schmidt A (2005). Safety and enhanced immunogenicity of a hepatitis B core particle plasmodium falciparum malaria vaccine formulated in adjuvant montanide ISA 720 in a phase I trial.. Infect Immun.

[33] Bottcher B, Wynne S, Crowther R (1997). Determination of the fold of the core protein of hepatitis B virus by electron cryomicroscopy.. Nature.

[34] Zlotnick A, Cheng N, Stahl S, Conway J, Steven A (1997). Localization of the C terminus of the assembly domain of hepatitis B virus capsid protein: Implications for morphogenesis and organization of encapsidated RNA.. Proc Natl Acad Sci U S A.

[35] Vanderberg J, Nussenzweig R, Most H (1969). Protective immunity produced by the injection of x-irradiated sporozoites of plasmodium berghei. V. in vitro effects of immune serum on sporozoites.. Mil Med.

[36] Persson C, Oliveira G, Sultan A, Bhanot P, V N (2002). Cutting edge: A new tool to evaluate human pre-erythrocytic malaria vaccines: Rodent parasites bearing a hybrid plasmodium falciparum circumsporozoite protein.. J Immunol.

[37] Kumar KA, Oliveira GA, Edelman R, Nardin E, Nussenzweig V (2004). Quantitative plasmodium sporozoite neutralization assay (TSNA).. J Immunol Methods.

[38] Nardin E, Calvo-Calle J, Oliveira G, Nussenzweig R, Schneider M (2001). A totally synthetic polyoxime malaria vaccine containing plasmodium falciparum B cell and universal T cell epitopes elicits immune responses in volunteers of diverse HLA types.. J Immunol.

[39] R Development Core Team (2007). R: A language and environment for statistical computing. Vienna, Austria: R Foundation for Statistical Computing.

[40] Yin JZ, Bell MK, Thorbecke GJ (1989). Effect of various adjuvants on the antibody response of mice to pneumococcal polysaccharides.. J Biol Response Mod.

[41] Wong VK, Quagliata R, Adler R, Kim KS (1991). Dose-related immunogenicity of haemophilus influenzae type b capsular polysaccharide-neisseria meningitidis outer membrane protein conjugate vaccine.. Am J Dis Child.

[42] Kotloff KL, Wasserman SS, Losonsky GA, Thomas W, Nichols R (2001). Safety and immunogenicity of increasing doses of a clostridium difficile toxoid vaccine administered to healthy adults.. Infect Immun.

[43] Schofield L, Villaquiran J, Ferreira A, Schellekens H, Nussenzweig R (1987). Gamma interferon, CD8+ T cells and antibodies required for immunity to malaria sporozoites.. Nature.

[44] Wang R, Richie TL, Baraceros MF, Rahardjo N, Gay T (2005). Boosting of DNA vaccine-elicited gamma interferon responses in humans by exposure to malaria parasites.. Infect Immun.

[45] Bomford R (1984). Relative adjuvant efficacy of al(OH)3 and saponin is related to the immunogenicity of the antigen.. Int Arch Allergy Appl Immunol.

[46] Ferreira A, Schofield L, Enea V, Schellekens H, van der Meide P (1986). Inhibition of development of exoerythrocytic forms of malaria parasites by gamma-interferon.. Science.

[47] Maheshwari RK, Czarniecki CW, Dutta GP, Puri SK, Dhawan BN (1986). Recombinant human gamma interferon inhibits simian malaria.. Infect Immun.

[48] de Koning-Ward TF, O'Donnell RA, Drew DR, Thomson R, Speed TP (2003). A new rodent model to assess blood stage immunity to the plasmodium falciparum antigen merozoite surface protein 119 reveals a protective role for invasion inhibitory antibodies.. J Exp Med.

[49] John CC, O'Donnell RA, Sumba PO, Moormann AM, de Koning-Ward TF (2004). Evidence that invasion-inhibitory antibodies specific for the 19-kDa fragment of merozoite surface protein-1 (MSP-1 19) can play a protective role against blood-stage plasmodium falciparum infection in individuals in a malaria endemic area of africa.. J Immunol.

[50] Edelman R, Hoffman S, Davis J, Beier M, Sztein M (1993). Long-term persistence of sterile immunity in a volunteer immunized with X-irradiated plasmodium falciparum sporozoites.. J Infect Dis.

[51] Gordon D, McGovern T, Krzych U, Cohen J, Schneider I (1995). Safety, immunogenicity, and efficacy of a recombinantly produced plasmodium falciparum circumsporozoite protein-hepatitis B surface antigen subunit vaccine.. J Infect Dis.

[52] Hollingdale MR, Nardin EH, Tharavanij S, Schwartz AL, Nussenzweig RS (1984). Inhibition of entry of plasmodium falciparum and P. vivax sporozoites into cultured cells; an in vitro assay of protective antibodies.. Journal of Immunology.

[53] Hoffman S, Wistar RJ, Ballou W, Hollingdale M, Wirtz R (1986). Immunity to malaria and naturally acquired antibodies to the circumsporozoite protein of plasmodium falciparum.. N Engl J Med.

[54] Wirtz RA, Ballou WR, Schneider I, Chedid L, Gross MJ (1987). Plasmodium falciparum: Immunogenicity of circumsporozoite protein constructs produced in escherichia coli.. Exp Parasitol.

[55] Hollingdale MR, Hogh B, Petersen E, Wirtz RA, Bjorkmann A (1989). Age-dependent occurrence of protective anti-plasmodium falciparum sporozoite antibodies in a holoendemic area of liberia.. Trans R Soc Trop Med Hyg.

[56] Hollingdale MR, Appiah A, Leland P, do Rosario VE, Mazier D (1990). Activity of human volunteer sera to candidate plasmodium falciparum circumsporozoite protein vaccines in the inhibition of sporozoite invasion assay of human hepatoma cells and hepatocytes.. Trans R Soc Trop Med Hyg.

[57] Schwenk R, Asher L, Chalom I, Lanar D, Sun P (2003). Opsonization by antigen-specific antibodies as a mechanism of protective immunity induced by plasmodium falciparum circumsporozoite protein-based vaccine.. Parasite Immunol.

