# Supplementary material for: Phase I Trial of an Alhydrogel Adjuvanted Hepatitis B Core Virus-Like Particle Containing Epitopes of Plasmodium falciparum Circumsporozoite Protein
Source: PLoS One. 2008 Feb 6;3(2):e1556. doi: 10.1371/journal.pone.0001556 (PMC2216688; doi:10.1371/journal.pone.0001556)
Supplement: Protocol S1 — Final Trial Protocol (0.78 MB PDF) [file pone.0001556.s002.pdf]

Phase I trial of ICC-1132, a candidate vaccine against *Plasmodium falciparum* malaria based on a viral-like particle comprising recombinant hepatitis B core antigen and circumsporozoite epitopes, to assess vaccine safety and immunogenicity in healthy adult volunteers

26th March 2004

Protocol Version Number	16
NIH Protocol Number	01-416
CVD Protocol Number	16000
UMB-BRAAN Number	H-21488
UMB-IRB Number	0402106
Apovia, Inc. Designation	ICC-1132, Malariavax

Principle Investigator

Robert Edelman, M.D.
Associate Director for Clinical Research
Professor of Medicine & Pediatrics
Center for Vaccine Development
University of Maryland School of Medicine
Baltimore, Maryland

Co-Investigators

Aric L. Gregson, M.D.
Vaccinology Fellow, Malaria Section
Center for Vaccine Development
University of Maryland School of Medicine
Baltimore, Maryland

Christopher V. Plowe, M.D., M.P.H.
Associate Professor of Medicine
Director, Malaria Section
Center for Vaccine Development
University of Maryland School of Medicine
Baltimore, Maryland

James Campbell, M.D.
Assistant Professor of Pediatrics
Division of Infectious Diseases & Tropical Pediatrics
Center for Vaccine Development
University of Maryland School of Medicine
Baltimore, Maryland

Karen Kotloff, M.D.
Professor of Pediatrics
Division of Infectious Diseases & Tropical Pediatrics
Center for Vaccine Development
University of Maryland School of Medicine
Baltimore, Maryland

Myron Levine, M.D., D.T.P.H.
Director, Center for Vaccine Development
Professor of Medicine & Pediatrics
University of Maryland School of Medicine
Baltimore, Maryland

Elizabeth Nardin, Ph.D.
Associate Professor
Department of Medical & Molecular Parasitology
The New York University School of Medicine
New York, New York

Ashley J. Birkett, Ph.D.
Director of Research
Apovia, Inc.
San Diego, California

NIAID Program Officer	B. Fenton Hall, M.D., Ph.D.<br>Chief, Malaria Vaccine Development Section<br>Parasitology & International Programs Branch<br>Division of Microbiology & Infectious Diseases<br>NIAID, National Institutes of Health<br>Bethesda, Maryland
IND Sponsor	National Institute of Allergy & Infectious Disease (NIAID)<br>Division of Microbiology & Infectious Diseases<br>NIAID, National Institutes of Health<br>Bethesda, Maryland
Corporate Sponsor	Apovia, Inc.<br>11125 Flintkote Avenue, Suite A<br>San Diego, California 92121
Independent Safety Monitor	Carol Tacket, M.D.<br>Professor of Medicine<br>Center for Vaccine Development<br>Division of Geographic Medicine<br>University of Maryland School of Medicine<br>Baltimore, Maryland

Safety Monitoring Committee	Carol Tacket, M.D.<br>Professor of Medicine<br>Center for Vaccine Development<br>Division of Geographic Medicine<br>University of Maryland School of Medicine<br>Baltimore, Maryland
	Wendy A. Keitel, M.D.<br>Associate Professor<br>Departments of Molecular Virology, Microbiology & Medicine<br>Baylor College of Medicine<br>Houston, Texas
	Alan McGill, M.D., FACP, COL, MC, U.S. Army<br>Science Director<br>Walter Reed Army Institute of Research<br>Walter Reed Army Medical Center<br>Washington, D.C. 20910-7500
Study Sites	Center for Vaccine Development Outpatient Unit<br>HSF Building I, Room 420<br>University of Maryland<br>Baltimore, Maryland
	General Clinical Research Center (GCRC)<br>University of Maryland Medical System Hospital<br>Baltimore, Maryland

Research Objectives

To assess and compare in a dose escalating study the safety and reactogenicity in healthy adult volunteers of up to 3 intramuscular injections of ICC-1132 adjuvanted with alhydrogel

To assess and compare in the same dose escalating study the immunogenicity of the above mentioned vaccine formulation

Volunteers Up to 80 healthy adults aged 18–45 years will be recruited, up to 52 will receive injections

Duration of Study One year (each volunteer)

Contents

1 Synopsis **8**

2 Introduction **8**
2.1 Background on Malaria . . . 8
2.1.1 Lifecycle of the Malaria Parasite . . . 9
2.2 Background on Malaria Sporozoite Vaccines . . . 9
2.2.1 Irradiated Sporozoites . . . 9
2.2.2 Circumsporozoite Vaccines . . . 10
2.2.3 Alum-Adsorbed Malaria Vaccines and Hypersensitivity Reactions . . . 11
2.3 ICC-1132 Antigen and Vaccine Experience . . . 11
2.3.1 Background Information . . . 11
2.3.2 Antigen Description . . . 11
2.3.3 Adjuvant Description . . . 12
2.3.4 Source of Vaccine . . . 12
2.3.5 Purification and Formulation . . . 12
2.3.6 Vaccine Stability . . . 12
2.4 Dosage Justification . . . 12
2.5 Animal Studies . . . 13
2.5.1 Rodents . . . 13
2.5.2 Primates . . . 15
2.5.3 Humans . . . 17

3 Study Objectives **18**

4 Institutional Review Boards **18**

5 Informed Consent **20**
5.1 Potential Benefits and Risks of Vaccination and Study Participation . . . 20
5.1.1 Potential Benefits . . . 20
5.1.2 Potential Risks . . . 20
5.1.3 Minimization of Risks . . . 21

6 Study Population **21**
6.1 Study Sites . . . 21
6.2 Inclusion Criteria . . . 21
6.3 Exclusion Criteria . . . 22

7 Study Plan **24**
7.1 Design . . . 24
7.1.1 Dosage Preparation . . . 25
7.1.2 Vaccination Process . . . 25
7.1.3 Post-Immunization Procedures . . . 25
7.1.4 Breaking the Study Blind . . . 26
7.2 Record Keeping and Monitoring . . . 26
7.3 Investigational Supplies . . . 26
7.3.1 Investigational Product Description . . . 26
7.3.2 Documentation of Vaccine Condition on Arrival at Study Site . . . 26
7.3.3 Storage Requirements . . . 26
7.3.4 Vaccine Accountability . . . 27
7.4 Study Methods . . . 27
7.4.1 Recruitment . . . 27
7.4.2 Screening and Enrollment Process . . . 27
7.4.3 Timing of Screening Labs . . . 28
7.4.4 Contraindications to Vaccination . . . 28
7.4.5 Indications for Deferral of Vaccination . . . 28
7.4.6 Detailed Description of Study Visits . . . 29
7.4.6.1 Study Day 0 (Baseline, Day of First Injection) . . . 29
7.4.6.2 Study Day 1 (24 ± 6 hours after First Injection) . . . 29
7.4.6.3 Study Day 2 (48 ± 6 hours after First Injection) . . . 29
7.4.6.4 Study Day 4 ± 1 . . . 29
7.4.6.5 Study Day 7 ± 1 . . . 30
7.4.6.6 Study Day 14 ± 2 . . . 30
7.4.6.7 Study Day 28 ± 4 . . . 30
7.4.6.8 Study Day 56 ± 4 (Day of Second Injection) . . . 30
7.4.6.9 Study Day 57 ± 4 (24 ± 6 hours after Second Injection) . . . 30
7.4.6.10 Study Day 58 ± 4 (48 ± 6 hours after Second Injection) . . . 31
7.4.6.11 Study Day 60 ± 4 (4 ± 1 days after Second Injection) . . . 31
7.4.6.12 Study Day 63 ± 5 (7 ± 1 days after Second Injection) . . . 31
7.4.6.13 Study Day 70 ± 6 (14 ± 2 days after Second Injection) . . . 31
7.4.6.14 Study Day 84 ± 8 (28 ± 4 days after Second Injection) . . . 31
7.4.6.15 Study Day 140 ± 11 (84 ± 7 days after Second Injection) . . . 31
7.4.6.16 Study Day 168 ± 14 (Day of Third Injection) . . . 32
7.4.6.17 Study Day 169 ± 14 (24 ± 6 hours after the Third Injection) . . . 32
7.4.6.18 Study Day 170 ± 14 (48 ± 6 hours after Third Injection) . . . 32
7.4.6.19 Study Day 172 ± 15 (4 ± 1 days after Third Injection) . . . 32
7.4.6.20 Study Day 175 ± 15 (7 ± 1 days after Third Injection) . . . 32
7.4.6.21 Study Day 182 ± 16 (14 ± 2 days after Third Injection) . . . 33
7.4.6.22 Study Day 196 ± 18 (28 ± 4 days after Third Injection) . . . 33
7.4.6.23 Study Day 224 ± 21 (56 ± 7 days after Third Injection) . . . 33
7.4.6.24 Study Day 336 ± 28 (168 ± 14 days after Third Injection) . . . 33
7.4.7 Volunteer Symptom Diary . . . 33
7.4.8 Laboratory Testing . . . 34
7.5 Volunteer Compensation . . . 34
7.6 Immunologic Assays . . . 34
7.6.1 Serologic Assays . . . 34

7.6.1.1 Antibody Avidity Assays . . . 35
7.6.2 Cellular Assays for Cell-Mediated Immunity (CMI) . . . 35

8 Adverse Events 35
8.1 Definitions . . . 36
8.2 Adverse Events Assessment . . . 36
8.2.1 Assessment of Intensity . . . 36
8.2.2 Assessment of Causality . . . 36
8.2.3 Pregnancy . . . 37
8.2.4 Treatment of Adverse Events . . . 37
8.3 Serious Adverse Events . . . 38
8.3.1 Definitions of Serious Adverse Events . . . 38
8.3.2 Reporting . . . 38
8.4 Adverse Events Monitoring . . . 38
8.4.1 Role of Independent Safety Monitor . . . 38
8.4.1.1 ISM Criteria to Suspend the Trial . . . 39
8.4.2 Role of Safety Monitoring Committee . . . 39
8.4.2.1 Analysis Plan for the SMC . . . 39
8.4.2.2 SMC Criteria to Suspend the Trial . . . 40
8.5 Criteria for Study Termination . . . 41

9 Analysis 41
9.1 Expected Outcome . . . 41
9.2 Sample Size Calculation . . . 41
9.3 Randomization Procedure . . . 41
9.4 Safety Analysis . . . 41
9.5 Immunologic Analysis . . . 42
9.5.1 Serologic Responses . . . 42
9.5.1.1 Antibody Avidity Responses . . . 42
9.5.2 Cellular Assays of CMI Responses . . . 42

10 Withdrawal from Study 43

11 References 43

12 Appendix A: Study Events Calendar 47

13 Appendix B: Volunteer Written Exam 47
13.1 Answer Key to Exam . . . 52

14 Appendix C: Adverse Events Grading 52
14.1 Abbreviations . . . 52
14.2 Estimating Severity Grade for Values Not in Tables, Symptom Diary or Clinical Evaluation Form . . . 53
14.3 DMID Toxicity Tables . . . 53
14.3.1 Hematology . . . 53
14.3.2 Chemistry and Urinalysis . . . 54
14.3.3 Cardiovascular . . . 54
14.3.4 Respiratory . . . 55

14.3.5 Gastrointestinal . . . 55
14.3.6 Neurological . . . 56
14.3.7 Musculoskeletal . . . 57
14.3.8 Skin . . . 57
14.3.9 Systemic . . . 58

15 Appendix D: Anaphylaxis SOP 58

16 Appendix E: Screening Exclusion Clinical Laboratory Values 59

17 Appendix F: Volunteer Recruitment Material 59
17.1 Recruitment Flyers . . . 59
17.2 Malaria Fact Sheet . . . 59
17.3 Information About Malaria Vaccine Sheet . . . 61
17.4 Overview of Each Study Visit and Estimated Time Required . . . 62

18 Appendix G: Continued Vaccination 62
18.0.1 Individual . . . 62
18.0.2 Cohort . . . 64

List of Figures

1 Dose-response after priming on days 21, 42 and boosting on day 56 (mice) . . . 14
2 Antibody response against the NANP epitope two weeks after the second administration (mice) 14
3 Isotype distribution of induced antibodies (mice) . . . 15
4 IFA versus ELISA (mice) . . . 16
5 Immunogenicity in rats . . . 16
6 Immunogenicity in rabbits . . . 17
7 Immune response in primates . . . 18

List of Tables

1 Human Studies: Cardiff Volunteers (first two rows) CVD Volunteers (last three rows) . . . 19
2 Study Events Calendar . . . 48
3 Visit Overview . . . 63

1 Synopsis

This is a phase I, dose-escalating clinical trial of a candidate malaria vaccine, ICC-1132. The primary objective is to assess and compare the safety, reactogenicity, and immunogenicity of three intramuscular injections of ICC-1132. The vaccine is adsorbed to alhydrogel adjuvant. Three dose levels, 10 μg, 20 μg and 50 μg, will be compared; each injected intramuscularly on study days 0, 56 $\pm$ 4 and 168 $\pm$ 14, with the exception of the 10 μg dose cohort which will receive only two injections, one each at 0 and 2 months. The vaccine is a viral-like particle produced in *Escherichia coli* (*E. coli*) and comprised of recombinant hepatitis B core protein into which B and T cell epitopes of the *Plasmodium falciparum* circumsporozoite protein (CSP) have been inserted. The B-cell epitopes include $(\text{NANP})_3$ and NANPNVDP sequences derived from the internal repeat region of CSP and the T-cell epitopes include the NANPNVDP sequence and a universal T-cell epitope derived from a non-repeat region of CSP.

The study was originally designed as a blinded, dose-escalating trial comparing three doses (10, 20 and 50 μg) of ICC-1132 in saline to three doses of ICC-1132 + alhydrogel (10, 20 and 50 μg). Sixteen volunteers in each of three cohorts were to be randomized to receive either of the two formulations, eight volunteers each. In January 2003, before recruitment into the 20 μg cohort completed, the trial was delayed due to stability problems with the saline formulation of the vaccine (see Section 2.3.6 on page 12). Because of these stability issues, the saline formulated vaccine was removed from the study.

Prior to removing the saline formulated ICC-1132 from the trial, the first 16 eligible volunteers were assigned to the 10 μg cohort, with eight receiving ICC-1132 in saline and eight ICC-1132 + alhydrogel. The next three eligible volunteers were assigned to the 20 μg cohort and were randomly assigned to receive ICC-1132 in saline or ICC-1132 + alhydrogel. Of the three volunteers in the first 20 μg cohort, two received one injection of the alhydrogel formulation and one received one injection of the saline formulation. These first 19 volunteers have been withdrawn from the study by the investigators and will receive no further vaccinations.

The study will continue with vaccinations *using only the alhydrogel formulation of the vaccine.* Because the dose schedule of the three volunteers in the original 20 μg dose cohort deviated markedly from the study protocol, it will not be possible to compare their reactogenicity and immunogenicity data with the other volunteers in the 10 and 50 μg dose cohorts. Therefore, in order to ensure parity between alum groups, eight new volunteers will enter the 20 μg dose cohort. The subsequent 25 eligible volunteers will be assigned to the 50 μg cohort and will receive three injections of ICC-1132 + alhydrogel.

Three immunizations of the assigned alhydrogel formulated vaccine dose will be administered to each volunteer on study days 0, 56 $\pm$ 4 and 168 $\pm$ 14.

2 Introduction

2.1 Background on Malaria

In most tropical and many subtropical developing countries, malaria is a major cause of morbidity, especially among young children and pregnant women, and mortality, principally among infants and toddlers (1). Malaria also represents a serious health hazard for travelers from industrialized countries that visit malaria-endemic areas. Worldwide, over 500 million infections and 2–3 million deaths are caused each year by malaria. The widespread resistance of *Anopheles spp.* mosquitoes to insecticides has crippled national and regional malaria control programs based on vector control interventions. Furthermore, the increasing prevalence of chloroquine and antifolate resistant strains of *Plasmodium falciparum* in Asia, Africa and South America has created a crisis in the clinical treatment of malaria in many countries. These drug resistant strains have made devising well-tolerated and effective prophylactic regimens challenging. Given these challenges, there

is an urgent need for new, effective interventions that can be applied to the control of malaria. One such approach involves immunoprophylaxis by means of malaria vaccines.

2.1.1 Lifecycle of the Malaria Parasite

Malaria parasites have a complex life cycle. Saliva from infected mosquitoes transmits the malaria sporozoites to man while the female mosquito is taking a blood meal. The sporozoites travel through the blood stream and invade hepatocytes where they mature into merozoites. After six to ten days, the infected hepatocytes rupture, releasing a large number of merozoites into the bloodstream. These merozoites then invade erythrocytes where they multiply and, after two days, release progeny merozoites. The progeny invade new erythrocytes to perpetuate the erythrocytic cycle. This cycle is responsible for clinical illness in humans. A small percentage of the merozoites do not multiply after invading erythrocytes, but instead differentiate into sexual forms called gametocytes. When ingested by a female mosquito, male and female sexual forms can unite, creating a zygote. The zygote matures and releases sporozoites, which migrate to the mosquito's salivary glands, thus completing the lifecycle.

2.2 Background on Malaria Sporozoite Vaccines

The sporozoite stage of *P. falciparum* is one potential target of a malaria vaccine. Erythrocyte-stage merozoites and gametocytes are other targets for vaccine development, but they will not be discussed further here. As described above, sporozoites are inoculated into the blood stream by the bite of an infected mosquito and invade hepatocytes within an hour. If immune mechanisms can prevent sporozoites from entering or developing within hepatocytes, the release of merozoites from the liver and resulting clinical malaria will thereby be averted. Thus, an effective sporozoite vaccine should stimulate the production of serum antibodies capable of neutralizing sporozoites before they can invade hepatocytes. Optimally, such a vaccine would also be recognized by T-cells, thereby generating immunologic memory capable of boosting the antibody response upon subsequent exposures to sporozoites (2;3). In addition, an ideal vaccine should sensitize cytotoxic T-cells and cytokine-producing T-cell subsets, because these T-cells interfere with the exo-erythrocytic cycle of parasite development (4).

2.2.1 Irradiated Sporozoites

Experiments with irradiated sporozoites in vitro and in rodent malaria models indicate that a variety of mechanisms participate in parasite killing and are important in the induction and maintenance of immunity to malaria (5). These mechanisms include antibody, gamma interferon, other cytokines, and direct cellular cytotoxicity. In rodent malaria models, the passive transfer of monoclonal antibody specific for the repeats of CSP can protect naive recipients against sporozoite challenge (6). The passive transfer of malaria-specific CD4+ T-cells can also protect naive mice against sporozoite challenge (7;8). Cytotoxic human CD4+ T-cell clones, derived from sporozoite-immunized volunteers, have been shown to lyse CSP peptide-coated target cells (9). In the early 1970's and 1990's, investigators at the University of Maryland, University of Illinois, and the U.S. military showed that significant protection could be elicited against experimental human malaria by immunizing volunteers with irradiated sporozoites (10-14). Such immunizing regimens involved the bites of hundreds of mosquitoes and did not represent a practical approach to vaccination. However, sera generated during these studies demonstrated that CSP was a critical antigen thought to be responsible for stimulating a protective immune response (15). Current biotechnology has made it possible to produce relevant CSP antigens in sufficient quantity to prepare synthetic and subunit vaccines suitable for clinical testing (16;17).

2.2.2 Circumsporozoite Vaccines

The first and most intensely studied antigens have been based on the CSP of *P. falciparum*. Several CSP antigens, produced through recombinant DNA technology and peptide synthesis, have been formulated into vaccines using various carriers and adjuvants. These products have been tested for safety, reactogenicity, immunogenicity, and efficacy in humans (18-25). Although these vaccines stimulated anti-sporozoite antibody production, the magnitude of the antibody responses was lower than expected based on animal studies, and not all vaccinees generated an immune response. Furthermore, the absence of "boosting" of antibody levels on subsequent injections and results of in vitro lymphocyte proliferation assays suggested that T-cells of most volunteers did not recognize the CSP antigen. Nevertheless, parasitemia was delayed or completely prevented in about 20% of vaccinees participating in these small efficacy studies (18-20;22;23).

In a major development 46 volunteers at Walter Reed were immunized with three formulations of a recombinant, hepatitis B surface antigen-CSP fusion vaccine (RTS,S) (25). Two of these formulations induced the development of high anti-CSP titers after two primary IM immunizations at zero and one months and a booster dose at six to seven months. Twenty-two of these vaccinees and six unimmunized control subjects were subsequently challenged with bites from *P. falciparum* infected mosquitoes. Six of seven volunteers who received RTS,S adjuvanted with monophosphoryl lipid A and QS-21 in an oil-in-water emulsion were protected from malaria infection (25). The longevity of the immune response was evaluated in immunized volunteers six months after the last vaccine dose and antibody levels decreased to between $\frac{1}{3}$ and $\frac{1}{2}$ of peak values (26). Seven previously protected volunteers were re-challenged with malaria-infected mosquitoes and two remained protected from infection. In a recent phase I/II study of 41 volunteers, RTS,S adjuvanted with monophosphoryl lipid A and QS-21 in an oil-in-water emulsion was found to have an overall protective efficacy of 41% (95% confidence interval, 22–56%) (27). Optimal protection against experimental infection occurred for the groups that received two or three standard dose or three intermediate strength doses.

Another malaria vaccine, SPf66, is a chemically synthesized peptide that contains amino acid sequences derived from three asexual-blood stage proteins linked by sequences derived from CSP. This vaccine was field-tested in South America (28), Africa (29;30), and Thailand (31) where it either did not protect, or it reduced episodes of clinical malaria by only 31–34%. When injected into Aotus monkeys, the SPf66 vaccine mixed with alum, QS-21 or Complete Freund's adjuvant protected 25%, 57% and 50% of animals, respectively, against *P. falciparum* challenge (R. Kammer, personal communication, by PSS).

While showing for the first time that safe and successful vaccination against the sporozoite stage of malaria is possible, these studies demonstrate the need for more immunogenic vaccine formulations before a malaria vaccine becomes a clinical reality. Evidence now indicates that both antibody and cell mediated mechanisms are operative in animals (4;5), but the role of CMI is unclear in humans. For example, in volunteers, *P. falciparum* CSP-specific cytotoxic T-lymphocytes in the peripheral blood induced by immunization with either irradiated sporozoites (12) or experimental CSP vaccines (23-25) are not strictly predictive of protection against experimental sporozoite challenge. By contrast, recent trials with the hepatitis B surface antigen-CSP fusion vaccine suggest that protection is associated with high titers of anti-CSP IFA antibody (25). The seven volunteers protected by immunization with irradiated sporozoites (12-14) all developed high anti-sporozoite IFA titers ranging from 1:1600-1:40,960. In mice there is a close correlation between the anti-sporozoite IFA titers engendered by multiantigen peptide (MAP) vaccines and resistance to sporozoite challenge (32;33). Although antibody alone might be sufficient to prevent infection, the degree to which anti-sporozoite antibody is, in vivo, functional and protective remains to be determined.

We believe that effective vaccines must be designed with a detailed knowledge of the ability of various epitopes within a given antigen to influence specific immune responses. This requires detailed studies to determine the extent to which various epitopes may induce, enhance, or indeed suppress, specific responses. An important aspect in elucidating the capability of an epitope will be the testing in humans of candidate vaccines of limited complexity and well-defined structure.

2.2.3 Alum-Adsorbed Malaria Vaccines and Hypersensitivity Reactions

Five investigational alum-adsorbed malaria vaccines—PfCS-MAP1NYU, SPf66, R32tet32, MSP-1, and Pfs25—have induced immediate-type hypersensitivity (ITH) reactions in human volunteers. In a phase I trial of the PfCS-MAP1NYU vaccine, two of 32 volunteers developed five to six pruritic wheals over their contralateral arm (the site of the vaccination given one month prior) 20–30 minutes after receiving their third injection (48). In addition, both volunteers developed generalized urticaria one to two hours after vaccination, without cardiovascular or respiratory compromise. While the systemic urticaria cleared after administration of oral anti-histamines medications, the pruritic swelling of the contralateral arm required five to seven days to resolve. In a phase I study of the SPf66 vaccine in 14 adult American volunteers, urticaria of the contralateral arm developed in two persons and contralateral arm urticaria plus systemic urticaria developed in one (24). In field studies of SPf66, contralateral arm and systemic urticaria developed in 0.3–1.5% of thousands of children and adults vaccinated in Africa, South America, and Thailand (29;31;34-37). One of 15 volunteers developed systemic urticaria after the third inoculation of R32tet32 (18). This volunteer was found to have detectable circulating anti-R32tet32 IgE. A fourth inoculation given three months later was non-reactive. In a phase I trial of a recombinant MSP-1 vaccine, three of 32 volunteers developed reactions: anaphylaxis occurred in one volunteer, generalized skin rash and contralateral arm inflammation occurred in another volunteer, and contralateral arm swelling in a third volunteer (38). The latter two reactions were not typical of ITH reactions. In an eight subject trial of recombinant gametocyte peptide (Pfs25), one volunteer developed contralateral urticaria (D.C. Kaslow, personal communication, by PSS).

The common features of these allergenic vaccines include 1) parenteral (intramuscular or subcutaneous) injections repeated two to three times at intervals of one month or more, 2) alternating injections between right and left arms, 3) formulation with alum or QS-21 adjuvant, 4) unbound antigen in the vaccine mixture, 5) relatively high mass of parasite-derived epitopes, 6) small peptides, and 7) high dose of antigen (200 to 2000 μg). Immediate-type hypersensitivity due to these vaccines occurred after the second, or more commonly, after the third vaccination administered one to five months after the second.

2.3 ICC-1132 Antigen and Vaccine Experience

2.3.1 Background Information

ICC-1132 is a viral-like particle produced in *E. coli* and comprised of recombinant hepatitis B core protein into which B and T-cell epitopes of *P. falciparum* CSP have been inserted. The viral-like particle forms spontaneously as a result of aggregation of the recombinant hepatitis B core proteins. The B-cell epitopes include $(\text{NANP})_n$ and NANPNVDP sequences derived from the internal repeat region of CSP; the T-cell epitopes include the NANPNVDP sequence and a universal T-cell epitope derived from a non-repeat region of the CSP.

2.3.2 Antigen Description

The antigen ICC-1132 comprises the hepatitis B core protein (149 amino acids) into which a 24-amino acid epitope has been inserted between position 78 and 79 of the native core protein. The inserted epitope comprises three repeats of the amino acid sequence N-A-N-P (asparagine-alanine-asparagine-proline) which is a conserved and protective B-cell epitope from the circumsporozoite protein, and a N-A-N-P-N-V-D-P sequence which comprises both a B-cell and T-cell epitope. As a result of the cloning procedure, the epitope is flanked by two amino acids each side. An additional 22 amino acid insertion at the end of the protein corresponds to a 20 amino acid universal T-cell epitope from CSP (326-345) and two amino acids that result from the cloning procedure. The universal T-cell epitope was identified from human volunteers who were protected against malaria after immunization with *P. falciparum* sporozoites (39;40). The universal T-cell epitope is associated with high cellular immune responses in human volunteers of diverse HLA types (41).

The resulting fusion protein self-assembles into a virus-like-particle with a size of roughly 30 nm diameter containing 240 copies of the ICC-1132 monomers.

2.3.3 Adjuvant Description

ICC-1132 will be formulated in the presence of alhydrogel. Alhydrogel is an aluminum hydroxide gel that has been widely used as an adjuvant in licensed vaccines. Previous versions of the protocol included a saline formulated ICC-1132. Because of problems with stability of the saline formulated ICC-1132, only alhydrogel formulated ICC-1132 will be used in the trial from from February 2003 onward (see Section 2.3.6).

2.3.4 Source of Vaccine

ICC-1132 is produced by recombinant technology. The gene encoding the protein is expressed in *E. coli.*

2.3.5 Purification and Formulation

E. coli expressing the gene coding for ICC-1132 are mechanically lysed and the ICC-1132 purified by ammonium sulphate precipitation. Successive gel exclusion chromatography, hydrophobic interaction chromatography, and hydroxyappatite chromatography is then undertaken. The resulting product is essentially free of contaminating *E. coli* proteins. In bulk, there is no more than 0.1 ng of DNA per 1 μg of ICC-1132. For alhydrogel formulated ICC-1132 the pyrogenicity USP < 151, which is within the limits for human use.

2.3.6 Vaccine Stability

The stability of both the saline and alhydrogel formulations was tested at 3, 6, 9 and 12 months. The available antigen in the saline formulated vaccine varied as much as 30% from vial to vial in the nine and 12 month stability assays. This could be prevented by adjustment of the salt concentration, modification of the pH, or the addition of mannitol. The study was already underway at the time of this discovery. As a result, we elected to discontinue the saline arm of the study in January 2003, because immunogenicity and reactogenicity data could not be accurately compared from vaccinee to vaccinee and between the two vaccine cohorts, saline versus alhydrogel formulations. A newly formulated saline vaccine will not be introduced into this trial. Alhydrogel formulated vaccine is stable, and will continue to be tested (B. Thornton, personal communication, February 5, 2003, by ALG).

2.4 Dosage Justification

The lowest dose, 10 μg, was chosen because this dose of ICC-1132 in saline is anticipated to induce a very low antibody titer. This conclusion is based on studies done in rhesus monkeys (see Section 2.5.2 on page 15 for review of primate studies). In these studies, a 20 μg dose of ICC-1132 in saline given on days 0 and 56 induced a low level immune response. Peak antibody titers of approximately 2,000 were achieved at day 90. By day 180, these titers dropped to below 100. Because antibody responses in humans are not likely to exceed that seen in monkeys, a dose of ICC-1132 in saline lower than or equal to 20 μg is likely to induce a low level antibody response. A dose of ICC-1132 in saline lower than 10 μg would present technical problems due to the limitation in sensitivity of the assays. Hence, 10 μg was chosen as the lowest dose.

The 20 and 50 μg doses are based on roughly two-fold increases in ICC-1132. In non-human primate studies, a 20 μg dose of ICC-1132 in saline appears to be sub-optimal. However, a 20 μg dose of ICC-1132 adjuvanted with alhydrogel appears optimal. It is an objective of the study to investigate the dose-response in humans.

The dosage schedule (days 0, 56 and 168) was selected based upon the Investigator's prior experiences with hepatitis B vaccines and animal studies (see Section 2.5) showing waning of antibody titer by nine weeks after a two doses of ICC-1132 in saline or adjuvanted with alhydrogel.

The third dose of the vaccine is important. With it, we will assess if a titer boost occurs in the ELISA anti-CSP or IFA anti-sporozoite antibody. Such a boost would reflect prior induction of immune memory, thought to be a critical component of the protective malaria immune response. Also, a third or booster injection will allow us to ascertain if antibody maturation occurs, reflected as increased anti-CSP antibody avidity. Enhanced avidity may be independent of the IFA or ELISA antibody titer. In fact, some experimental models would suggest antibody avidity may be as important, or more important than antibody titer in providing protection against malaria and other pathogens (42;43).

There is the potential issue of enhanced reactogenicity after the third injection associated with high titers of pre-existing antibody (see Section 2.2.3 on page 11). For example, in a phase I trial of the PfCS-MAP1NYU vaccine, persons with high antibody titers were more likely to have local inflammatory reactions at the vaccination site than persons with low antibody titers (48). Local inflammatory reactions included pain, tenderness, redness, and swelling, but there was no limitation of activity, particularly motion of the affected arm. Importantly, in this same study, no increase in systemic reactions was seen between volunteers with high and low antibody titers after receipt of the third dose of vaccine. These reasons (involving the promise of enhanced immunogenicity without clinically important reactogenicity) justify a third dose of vaccine.

2.5 Animal Studies

A significant number of immunogenicity and safety studies have been performed with ICC-1132 in saline and ICC-1132 adjuvanted with alhydrogel in animals.

2.5.1 Rodents

The dose response of ICC-1132 in saline was evaluated in mice (study number AJB-00-02). Mice (Balb/C) received intra-peritoneal injections with 0.04, 0.2, 1, 5 or 25 μg of ICC-1132 in saline at 0 and 42 days. The anti-(NANP)$_3$ titers were evaluated by ELISA. Titers were expressed as end point titers, which is determined as the last sequential dilution of the serum in the ELISA assay that gives an optical density (OD) greater than twice the background. The graph demonstrates the dose-response after priming on days 21 and 42 and boosting on day 56. (see figure 1 on the next page)

The immunogenicity of ICC-1132 in saline and ICC-1132 adjuvanted with alhydrogel was studied in mice (study number AJB-00-11). Mice (Balb/C, n=6) received 20 μg of ICC-1132 in saline or ICC-1132 adjuvanted with alhydrogel via intraperitoneal injection. The administration was repeated after four weeks. Below, the antibody response against the NANP epitope two weeks after the second administration is shown. These data indicate that alhydrogel augments the immune response against the ICC-1132 vaccine in mice. (see figure 2 on the following page)

The isotype distribution of antibodies induced in the above study (study number AJB-00-11) is also presented. (see figure 3 on page 15). For this study, pooled sera were used to evaluate isotype titers, hence no standard deviation can be calculated.

Alhydrogel-formulated ICC-1132 induces an immune response that is more biased towards IgG1, than is a saline-formulated ICC-1132 induced immune response. This is in accordance with current immunological teaching that aluminum-based formulations induce Th2 biased responses (primarily IgG1) in mice.

Because *P. falciparum* only infects humans, in vivo protection studies are very difficult to perform with *P. falciparum*. As an indirect measure of vaccine efficacy, the binding of antibodies induced by ICC-1132 in saline

Figure 1: Dose-response after priming on days 21, 42 and boosting on day 56 (mice)

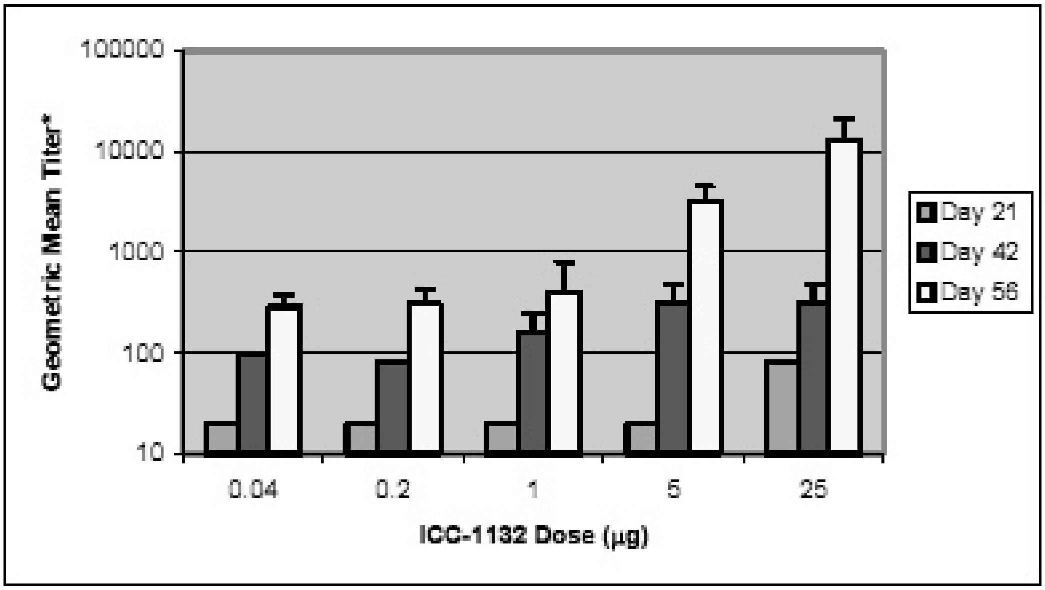

Figure 2: Antibody response against the NANP epitope two weeks after the second administration (mice)

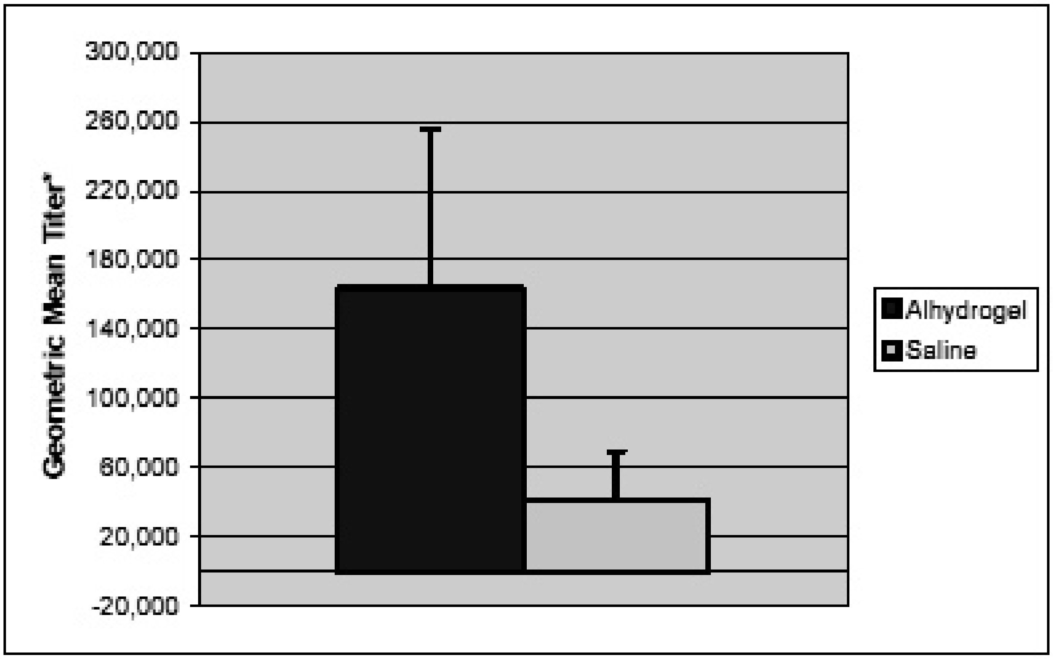

Figure 3: Isotype distribution of induced antibodies (mice)

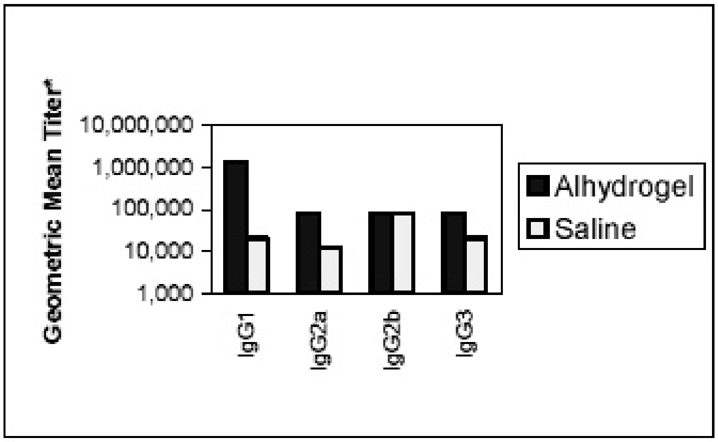

to *P. falciparum* sporozoites was evaluated (study number AJB-00-05). The IFA assay involves incubating sporozoites with sera and then detecting the bound antibodies with fluorescent anti-mouse antibodies.

Mice were injected via the intraperitoneal route with 20 μg ICC-1132 in saline at 0, 4 and 8 weeks. The sera from bleeds taken two weeks after the first injection (post 1st), two weeks after the second injection (post 2nd) and two weeks after the third injection (post 3rd) were evaluated for anti-NANP antibodies using a capture ELISA and for sporozoite binding capacity by IFA. The results demonstrate that the antibodies induced are capable of binding to the parasite. (see figure 4 on the following page)

ICC-1132 formulated in saline and ICC-1132 adjuvanted with alhydrogel has been evaluated for safety in both rats and rabbits. Rats were given three intramuscular injections of 0.1 ml of ICC-1132 in saline (18 μg dose) or 0.1 ml of ICC-1132 plus alhydrogel (4 or 18 μg dose). Injections were given at one month intervals on days 0, 30 and 60. Rabbits received three intramuscular injections of 0.5 ml of ICC-1132 in saline (90 μg dose) or 0.5 ml of ICC-1132 plus alhydrogel (20 or 90 μg dose). Injections were again given at one month intervals on days 0, 30 and 60. Both rats and rabbits were monitored for temperature change, weight loss, and changes in hematology and chemistry parameters. Changes in macroscopic and microscopic histopathology were also assessed.

Transient inflammation and reactogenicity typical of intramuscular injection was observed in some animals following three intramuscular doses in rats and rabbits. The lack of test article related toxicity was evident in every parameter evaluated in the study.

Evaluation of antibody titers in the study rats and rabbits demonstrate that ICC-1132 is immunogenic at both the low and the high dose. An antibody response was induced after the first administration of either ICC-1132 formulation and this response was boosted by subsequent vaccinations. In these animal models, alhydrogel enhanced the immune response. The serology data for rats and rabbits is shown in figure 5 on the next page and figure 6 on page 17, respectively.

2.5.2 Primates

Rhesus monkeys have been vaccinated with ICC-1132 formulated in saline or with alhydrogel. Animals received 20 or 90 μg of ICC-1132 in saline or with alhydrogel twice (on days 0 and 56) by intramuscular injection. Animals were monitored for clinical symptoms, as well as changes in hematology and blood chemistry parameters. No toxicological consequences were observed. Transient local reactogenicity was similar to that reported in humans for several alum-adjuvanted vaccines. No evidence of severe reactogenicity

Figure 4: IFA versus ELISA (mice)

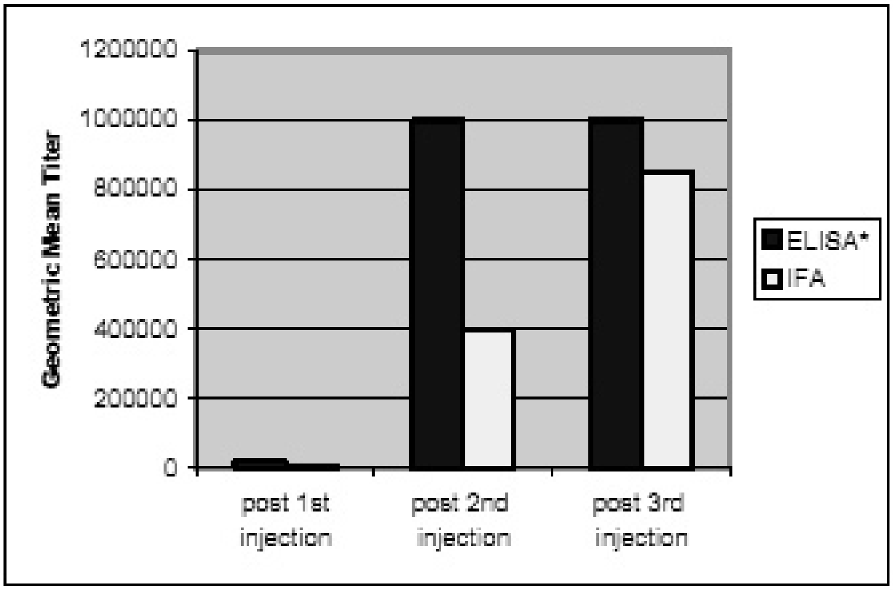

Figure 5: Immunogenicity in rats

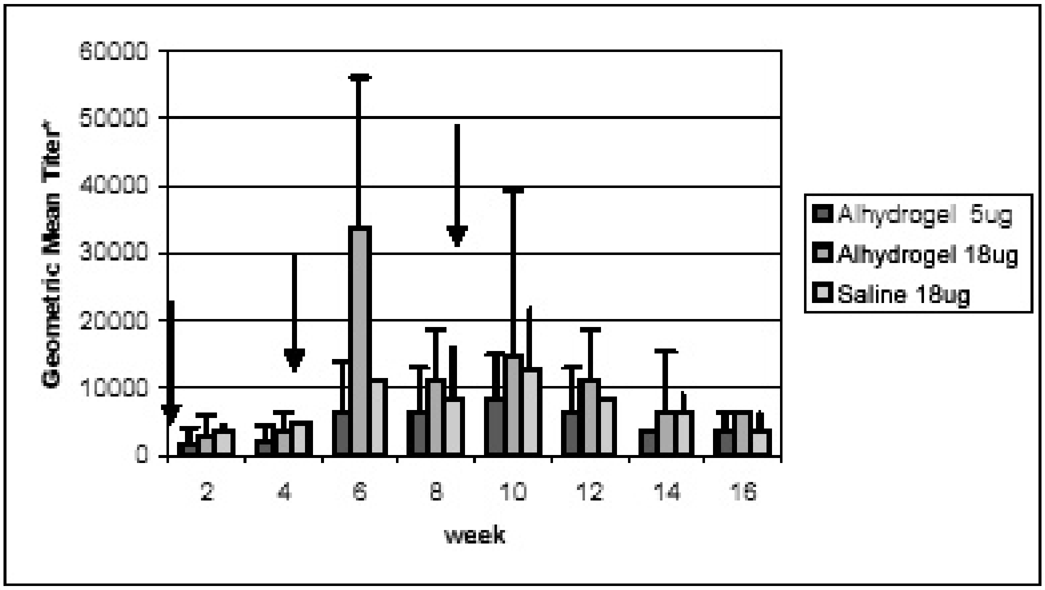

Figure 6: Immunogenicity in rabbits

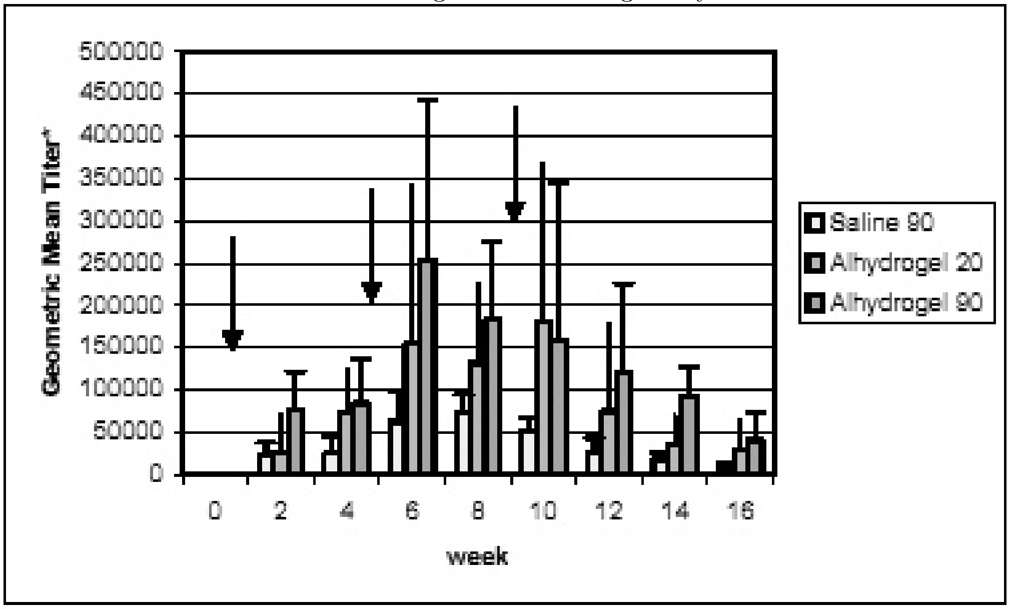

was observed in any animal at any time point. An immune response was induced by both formulations of ICC-1132 after the first vaccination. This response was boosted by the second vaccination. A graphical depiction of the immune response data is presented in figure 7 on the next page. This graph demonstrates that alhydrogel significantly enhances the immune response to ICC-1132 (49).

2.5.3 Humans

Three studies have evaluated the safety, reactogenicity and immunogenicity of ICC-1132 in humans. The first study evaluated ICC-1132 formulated in alhydrogel in Cardiff, Wales. Three doses of 20 μg of vaccine were administered to ten volunteers and three doses of 50 μg of vaccine were administered to eight volunteers using the same administration schedule as the present study. After twelve months of follow-up there were no serious adverse events and the vaccine was well tolerated at these dose levels. The most common adverse reaction was mild pain at the injection site.

A second, limited study in Tubingen, Germany evaluated the safety and immunogenicity of ICC-1132 formulated in Seppic ISA 720, a metabolizable oil adjuvant. A single dose of 5, 20 or 50 μg of vaccine was administered to each group of eight volunteers with two saline controls in each dose cohort. The volunteers were followed for 168 days post-vaccination. There were no serious adverse events reported. Anti-sporozoite and anti-hepatitis antibodies were detected 14–28 days post-vaccination, as was proliferation of and γ-interferon production by PBL of volunteers when stimulated with recombinant CS protein and ICC-1132 antigen (50).

The current study described within this protocol is the third study to use ICC-1132 in humans. This study has thus far evaluated the safety and reactogenicity of ICC-1132 in both saline and alhydrogel at the 10 and 20 μg dose levels in a total of 19 volunteers. Thirty-four injections were administered to the 19 volunteers, 31 injections at the 10 μg dose level and three at the 20 μg dose level. After more than six months of follow-up there were no serious systemic, local or laboratory adverse events related to vaccination. The

Figure 7: Immune response in primates

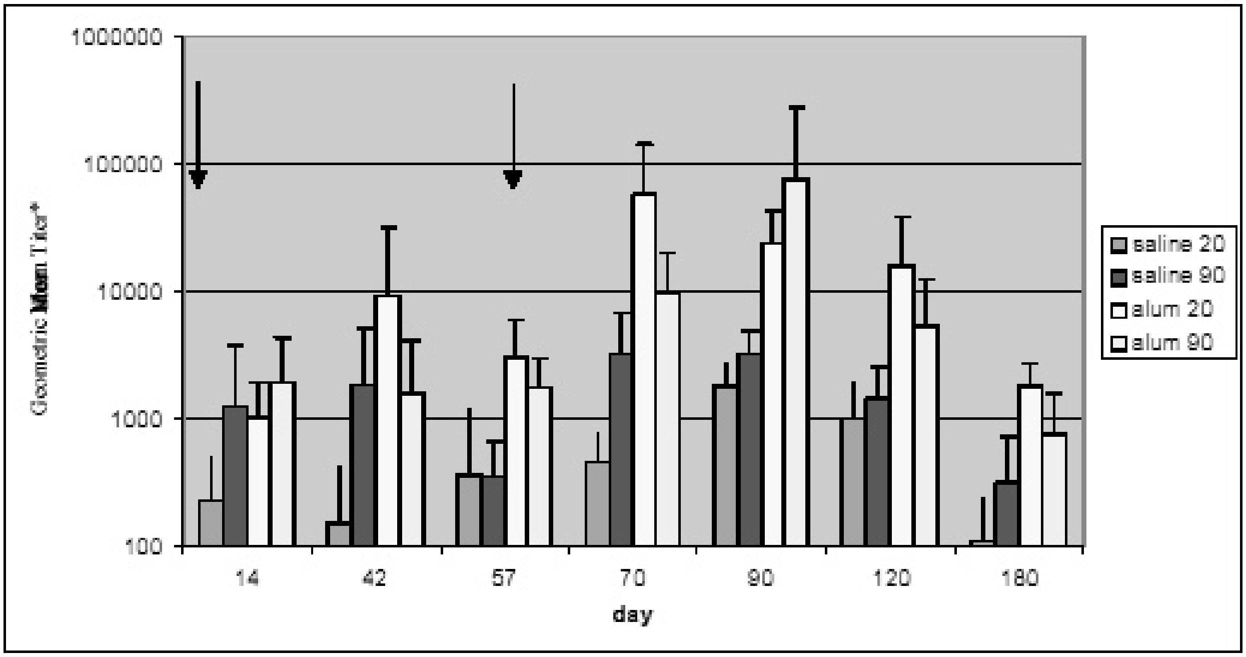

vaccine was well tolerated by the volunteers, regardless of dose or formulation. The most common adverse reaction was mild local tenderness or pain at the injection site. No volunteer experienced pruritus, erythema or induration at the injection site.

Immunogenicity data is yet incomplete for the T-cell responses, but the preliminary humoral data is encouraging, particularly from the current CVD study. Table 1 on the following page demonstrates the ELISA data from the Cardiff volunteers 28 days after their third vaccination and from the CVD volunteers 28 days after their second vaccination.

3 Study Objectives

1. To assess and compare in a dose escalating study the safety and reactogenicity in healthy adult volunteers of up to three intramuscular injections of ICC-1132 adjuvanted with alhydrogel.

2. To assess and compare in the same dose escalating study the immunogenicity of the above mentioned vaccine formulation.

4 Institutional Review Boards

Institutional Review Board approval of the protocol, informed consent document, advertisements, and amendments to these, will be obtained from a properly constituted IRB for the University of Maryland, Baltimore and the approval committee of the GCRC. Written approval of the IRB response will be submitted with the protocol, informed consent and advertising documents to the NIH Project Officer for approval.

Table 1: Human Studies: Cardiff Volunteers (first two rows) CVD Volunteers (last three rows)

Antigen Dose	Adjuvant	Number of Injections	(T1B)4 % positive (N) GMT	ICC-1132 % positive (N) GMT	HBc-149 % positive (N) GMT
20 μg	Alhydrogel	3	40 (4/10) 538 (160-2560)	100 (10/10) 2389 (320-40960)	90 (9/10) 1613 (320-20480)
50 μg	Alhydrogel	3	75 (6/8) 508 (160-2560)	100 (10/10) 2153 (320-20480)	88 (7/8) 2100 (320-40960)
10 μg	Saline	2	38 (3/8) 508 (320-1280)	50 (4/8) 453 (320-1280)	63 (5/8) 320 (160-640)
10 μg	Alhydrogel	2	100 (8/8) 640 (160-5120)	100 (8/8) 20480 (5120-81920)	100 (8/8) 13280 (640-163840)
20 μg	Saline	1	0 (0/1) 0	0 (0/1) 0	0 (0/1) 0
20 μg	Alhydrogel	1	0 (0/1) 0	100 (2/2) 160	50 (1/2) 80

5 Informed Consent

Before enrollment, screening is undertaken, with the screening process reviewed in detail with each prospective volunteer to answer questions about the screening process. HIPAA regulations will be followed and a HIPAA Consent will be signed by each volunteer prior to signing the screening consent and enrollment in the trial. If the volunteer agrees to undergo screening, a separate Screening Consent form is signed. This Screening Consent form was approved by the University of Maryland, Baltimore IRB and the approval committee of the GCRC. Before HIV testing, a part of the screening process, a State of Maryland Department of Health and Mental Hygiene AIDS Administration Informed Consent and Agreement to HIV Testing form must be reviewed and signed by the volunteer. Before enrollment in the study, defined as signing of the Research Consent form, volunteers are given a copy of the Research Consent form to read. In addition, the Principal Investigator or a designee gives a verbal description of the study to each potential volunteer. This may be done in a group setting or individually. This description often precedes the detailing of the screening process. Participants are given an opportunity to ask questions concerning the study and if they agree to participate, are required to sign a Research Consent form. Before enrollment in the study, each potential subject will be fully informed so that they understand the following:

- Participation in the study is entirely voluntary
- Refusal to participate involves no penalty or loss of medical benefits
- They may withdraw from the study at any time
- They are free to ask questions at any time to allow him or her to understand the purpose of the study

Each subject's understanding of the study protocol is evaluated by administering a 21 question written exam about the study. Subjects must score 70% or better prior to signing the Research Consent form.

5.1 Potential Benefits and Risks of Vaccination and Study Participation

5.1.1 Potential Benefits

Volunteers will not benefit directly from participation in this study. It is hoped that the information gained from this study will aid development of a safe and effective malaria vaccine.

5.1.2 Potential Risks

The general risks to participants in this phase I study are associated with phlebotomy and with vaccination. The volume (~860 ml) of blood drawn over the nine months of the study should not compromise these otherwise healthy subjects. This volume of blood is less than that found in two standard units of whole blood (each standard unit consists of 450 ml) and is within American Red Cross recommended blood donation limits. Potential risks include the following:

Local reactions: Tenderness, bruising, or fainting may result from blood draws. An inflammatory reaction (redness, swelling, tenderness and, rarely, infection) may occur at the site of injection.

Subcutaneous nodule formation: If the vaccine is given subcutaneously (under the skin), a nodule can form. These nodules are reactions to the adjuvant. These nodules can breakdown and form a sterile abscess or draining sore (pus pocket). Because of this possibility, injections will be given into the deltoid muscle of the arm.

Systemic Reactions: Systemic reactions such as a flu-like illness with low-grade fever, chills and malaise can occur. In studies of other malaria vaccines, these reactions resolved in 1–2 days without therapy. More severe reactions could, in theory, develop. These reactions could damage organs such as the liver or kidney. Temporary paralysis (known as Guillain-Barré syndrome) is a rare and potentially fatal condition that may occur with any vaccine. No case of Guillain-Barré syndrome has ever been associated with a malaria vaccine. Nevertheless, we cannot totally exclude the possibility that you might suffer such a paralysis. Most people recover from this condition, but some do not.

Allergic Reactions and Anaphylaxis: As with any vaccine, allergic reactions are possible. Anaphylaxis, a severe allergic reaction that may even be fatal, occurs rarely. This reaction may cause hives, itching, wheezing or trouble breathing. If anaphylaxis occurs, it almost always does so within minutes of vaccination. If anaphylaxis occurs, it may require treatment with injections of epinephrine, antihistamines, and/or steroids. In some cases, it requires the use of artificial respiration. The chances of such a severe reaction occurring are very small. Emergency medical care is immediately available just in case. You will need to remain in the clinic for 30 minutes after vaccination, so that we can watch you for signs of anaphylaxis.

Adventitious Agents: The study vaccine has been extensively tested for microbes and germs. It is free of known microbes that could contaminate the vaccine and infect humans. Nevertheless, there is a remote risk of getting an undetected microbe from the vaccine that could result in serious illness.

Positive hepatitis B core serology: The vaccine may induce antibodies to hepatitis B core antigen. Testing positive for hepatitis B core antibodies may make you ineligible for blood or organ donation. Antibodies to hepatitis B core antigen are not likely to be harmful. In fact, these antibodies may provide some protection against hepatitis B infection. At the beginning and end of the study, you will be tested for hepatitis B core antibodies, hepatitis B surface antigen and hepatitis B surface antibodies. If you test positive after getting the vaccine, you will be given a letter of explanation. This letter will say that you were vaccinated with an experimental malaria vaccine and that this was the probable cause of your positive hepatitis B core serology.

5.1.3 Minimization of Risks

As outlined above, the volunteers will be monitored closely during their participation in this study. The study vaccine has been prepared according to Good Manufacturing Procedures (GMP). The vaccine will be administered by experienced investigators with drugs and equipment available for the treatment of anaphylaxis. All vaccine doses will be given by intramuscular injection to minimize injection site reactions.

6 Study Population

6.1 Study Sites

1. Center for Vaccine Development Outpatient Unit, HSF Building 1, Room 420, University of Maryland Baltimore, Maryland

2. General Clinical Research Center (GCRC), University of Maryland Medical Center, Baltimore, Maryland

6.2 Inclusion Criteria

Subjects must **fulfill all** of the following criteria to be enrolled in this study:

- Male or female 18 to 45 years of age
- Willingness to participate in this study as evidenced by a signed, written informed consent
- An informed consent written exam score of at least 70%
- If female, willingness to avoid pregnancy and practice adequate birth control from the time of study enrollment until at least 2 months after the third vaccination
- Agrees to refrain from blood donation during the course of the study
- Agrees to be available for all scheduled study visits (vaccinations and follow-up)
- Agrees not to participate in concurrent vaccine or drug trials other than those evaluating Apovia's ICC-1132

6.3 Exclusion Criteria

Subjects will **not** be enrolled in this study if **any** of the following criteria are met:

1. Evidence of renal disease, as indicated by any of the following:
    - Creatinine > 1.5 mg/dL within the 7 days before first vaccination
    - RBC or WBC casts in urine
    - Urine protein ≥1+ on urinalysis
2. Evidence of cardiovascular disease, as indicated by any of the following:
    - BP > 150/90 mmHg in two measurements on different days
    - Hospitalization for heart attack, arrhythmia, or syncope
    - Murmur (other than a functional murmur) detected on physical examination
3. History of cancer (except basal call carcinoma of the skin and cervical carcinoma in situ)
4. Evidence of liver or other reticuloendothelial disease, as indicated by any of the following:
    - Positive serology for hepatitis B surface antigen
    - Positive serology for hepatitis C antibody
    - AST or ALT more than 1.5 times normal within the 7 days before first vaccination
    - Hepatosplenomegaly, jaundice, or lymphadenopathy on physical examination
5. Evidence of neurological disease, as indicated by any of the following:
    - History of seizures (other than febrile seizures as a child < 5 years old)
    - History of unconsciousness (other than a single brief "concussion")
    - Recurrent severe headaches or a diagnosis of migraine headaches
    - Focal neurological deficit on physical examination suggesting a pathologic process
6. Evidence of gastrointestinal disease, as indicated by any of the following:

- Recurrent diarrhea (> 5 episodes during the past 6 months, each lasting at least 3 days, with at least one week between episodes)
- Frequent indigestion or heartburn that requires daily antacids or other medical therapy
- Diagnosed by a doctor as having uncontrolled irritable bowel syndrome, Crohn's disease, ulcerative colitis, celiac disease, or stomach or intestinal ulcers
- Blood in the stool during the past year (other than occasional small amount from straining or hemorrhoids)

7. Evidence of hematologic, rheumatologic, or immunologic disease, as indicated by any of the following:
   - WBC < 3.0 x $10^3/mm^3$ or > 13.5 x $10^3/mm^3$ within the 7 days before first vaccination
   - Absolute neutrophil count < $1500/mm^3$ within the 7 days before first vaccination
   - Hemoglobin (within the 7 days before first vaccination)
     - Females < 10.5 g/dL or > 18 g/dL
     - Males < 11.5 g/dL or > 20 g/dL
   - History of ≥ 2 hospitalizations for invasive bacterial infections (pneumonia, meningitis)
   - History of hemoglobinopathy such as sickle cell disease or thalassemia
   - Diagnosis of collagen vascular disease such as lupus or dermatomyositis
   - Positive serology for HIV antibody
8. History of diabetes mellitus or a 3-hour fasting blood glucose > 125 mg/dL
9. Evidence of pulmonary disease as indicated by any of the following:
   - History of asthma requiring the use of oral medications or metered dose inhalers in the previous 12 months
   - Wheezes, rales, or prolonged expiratory phase on auscultation of the lungs
10. Is required to take a daily medication other than vitamins, levothyroxine, birth control pills, hormone replacement therapy for menopause, or the following medications for attention deficit hyperactivity disorder (pemoline [Cylert], methylphenidate HCl [Ritalin, Ritalin-SR, Concerta], dextroamphetamine sulfate [Dexedrine, Adderall], bupropion HCl [Wellbutrin, Wellbutrin-SR])
11. Receives allergy shots or uses allergy medications chronically
12. Behavioral, cognitive, or psychiatric disease that in the opinion of the investigator affects the ability of the volunteer to understand and cooperate with the study protocol
13. Volunteer has had medical, occupational, or family problems as a result of alcohol or illicit drug use during the past 12 months
14. Pregnancy (positive urine pregnancy test immediately prior to each dose, or positive serum pregnancy test during screening) or breastfeeding
15. Temperature > 38 °C (100.4 °F) or symptoms of an acute self-limited illness such as an upper respiratory infection or gastroenteritis on vaccination day; subjects may be rescheduled to enter the trial after illness has resolved, as per protocol

16. Use of investigational drugs, products, or devices within 30 days prior to study drug administration
17. Vaccination with live vaccine within 30 days or killed vaccine within 2 weeks
18. History of malaria infection or vaccination with candidate malaria vaccine
19. Allergy to aminoglycosides, tetracycline, or related antibiotics such as gentamicin, kanamycin or doxycycline
20. Weight less than 110 pounds
21. Other condition that in the opinion of the investigator would jeopardize the safety or rights of a volunteer participating in the trial or would render the subject unable to comply with the protocol
22. History of immediate-type hypersensitivity reaction to any vaccine

7 Study Plan

7.1 Design

This is a phase I, dose-escalating clinical trial of a candidate malaria vaccine, ICC-1132. The primary objective is to assess and compare the safety, reactogenicity, and immunogenicity of three intramuscular injections of ICC-1132. The vaccine is adsorbed to alhydrogel adjuvant. Three dose levels, 10 μg, 20 μg and 50 μg, will be compared; each injected intramuscularly on study days 0, 56 $\pm$ 4 and 168 $\pm$ 14, with the exception of the 10 μg dose cohort which will receive only two injections, one each at 0 and 2 months. The vaccine is a viral-like particle produced in *E. coli* and comprised of recombinant hepatitis B core protein into which B and T cell epitopes of the *P. falciparum* circumsporozoite protein (CSP) have been inserted. The B-cell epitopes include $(\text{NANP})_3$ and NANPNVDP sequences derived from the internal repeat region of CSP and the T-cell epitopes include the NANPNVDP sequence and a universal T-cell epitope derived from a non-repeat region of CSP.

The study was originally designed as a blinded, dose-escalating trial comparing three doses (10, 20 and 50 μg) of ICC-1132 in saline to three doses of ICC-1132 + alhydrogel (10, 20 and 50 μg). Sixteen volunteers in each of three cohorts were to be randomized to receive either of the two formulations, eight volunteers each. In January 2003, before recruitment into the 20 μg cohort completed, the trial was delayed due to stability problems with the saline formulation of the vaccine (see Section 2.3.6 on page 12). Because of these stability issues, the saline formulated vaccine was removed from the study.

Prior to removing the saline formulated ICC-1132 from the trial, the first 16 eligible volunteers were assigned to the 10 μg cohort, with eight receiving ICC-1132 in saline and eight ICC-1132 + alhydrogel. The next three eligible volunteers were assigned to the 20 μg cohort and were randomly assigned to receive ICC-1132 in saline or ICC-1132 + alhydrogel. Of the three volunteers in the first 20 μg cohort, two received one injection of the alhydrogel formulation and one received one injection of the saline formulation. These first 19 volunteers have been withdrawn from the study by the Investigators and will receive no further vaccinations.

The study will continue with vaccinations *using only the alhydrogel formulation of the vaccine*. Because the dose schedule of the three volunteers in the original 20 μg dose cohort deviated markedly from the study protocol, it will not be possible to compare their reactogenicity and immunogenicity data with the other volunteers in the 10 and 50 μg dose cohorts. Therefore, in order to ensure parity between alum groups, eight new volunteers will enter the 20 μg dose cohort. The subsequent 25 eligible volunteers will be assigned to the 50 μg cohort and will receive three injections of ICC-1132 + alhydrogel.

Three immunizations of the assigned alhydrogel formulated vaccine dose will be administered to each volunteer on study days 0, 56 ± 4 and 168 ± 14. Vaccinations will take place at the GCRC unit at the University of Maryland Medical System hospital in Baltimore.

Immunization of the 50 μg cohort will not begin until the Safety Monitoring Committee has reviewed the 14 day safety data following the first vaccination of volunteers in the 20 μg dose cohort and has determined that the vaccine reactions, if any, and clinical laboratory results are acceptable as outlined in Section 8.4.2 on page 39.

7.1.1 Dosage Preparation

A product temperature log will be maintained during vaccination days. Vials should be removed from the refrigerator and may be allowed to warm to room temperature for up to two hours prior to administration. Vials should be shaken gently and visually observed to be a homogeneous off-white to grayish white turbid liquid free of foreign particulate matter immediately prior to use. Once the vaccine is drawn into the syringe, it can be stored in the syringe up to one hour, *but* in the event of storage in the syringe beyond a few minutes (~ 5 minutes) the product *must* be remixed by tapping until it is homogeneous to visual observation.

7.1.2 Vaccination Process

On vaccination days, criteria for continued eligibility will be reviewed and verified, for both the individual and the cohort (see Section 18 on page 62). A history-directed physical examination will be done and oral temperature, blood pressure, pulse, respiratory rate and baseline general symptoms will be recorded. Venous blood will be collected for laboratory analysis as detailed in Section 12 on page 47.

One-half milliliter of ICC-1132 + alhydrogel (containing 0.5 mg of aluminum) will be administered intramuscularly into the deltoid muscle. The first and third immunizations will be given in the volunteer's non-dominant arm and the second immunization will be given in the dominant arm. If any local impairment prevents administration of the vaccine dose into the upper arm for that particular dose, the vaccine may be administered into the opposite arm in the deltoid region.

At the time of each injection an Investigator will be present. A Basic Life Support (BLS) trained health care professional and a standard emergency kit (crash cart) will be maintained in readiness on the trial site at these times. Anaphylaxis will be managed according to the Standard Operating Procedure (SOP) as detailed in Section 15 on page 58.

7.1.3 Post-Immunization Procedures

Subjects will be observed for immediate localized or systemic reactions for 30 minutes before being released from the clinic. Vital signs and a post-vaccination arm check will be performed approximately 30 minutes after vaccine administration.

Subjects will return to the outpatient clinic for clinical examination at 24 ± 6 and 48 ± 6 hours, and at days 7 ± 1, 14 ± 2, and 28 ± 4 after each vaccination, as detailed in Section 7.4.6 on page 29. Clinical exam will include an interval history of systemic and local reactions, examination of the site of injection and an oral temperature. Local injection site parameters to be recorded include erythema and induration (in mm), local (spontaneous) pain, tenderness (pain to touch), limitation of arm movement, and subcutaneous nodule formation. Examination for axillary node tenderness will be conducted if there is limited arm motion. Local reactions will be graded as detailed in Sections 7.4.7 on page 33 and 14 on page 52.

Volunteers will complete a daily symptom diary for seven days after each vaccination. Additional follow up visits will be done 84 ± 7 days after the second vaccine and 56 ± 7 days after the third vaccine. A telephone interview will be done at day 4 ± 1 after each immunization and 168 ± 14 days after the third immunization (see Section 12 on page 47).

The Study Events Calendar is detailed in Section 12 on page 47.

7.1.4 Breaking the Study Blind

The study investigators were blinded to the vaccine formulation, saline vs. alhydrogel, given to volunteers in the 10 μg cohort and the first three volunteers in the 20 μg cohort. These volunteers' study randomization codes were unblinded for safety purposes. Unblinding was discussed with the sponsors, the Principle Investigator, the Senior Co-Investigators, the Local Medical Monitor, the Safety Monitoring Committee and the DMID.

7.2 Record Keeping and Monitoring

Overall conduct of the study including the recruitment and acceptance of subjects, data collection, the proper recording of data, submission of reports and ultimate publication of the results, is the responsibility of the Principal and Co-Investigators. With the exception of laboratory test results, the Case Report Form (CRF) will serve as the source document for data collected in this trial. The Principal Investigator will report all Serious Adverse Events (SAE) to the Institutional Review Board and to the NIAID. All grade 3 or 4 adverse events will be reported to the NIAID. Data analysis and preparation of the results for publication in scientific literature will be the primary responsibility of the Principal Investigator and Co-Investigators.

Scheduling protocol events such as ICC-1132 administration, obtaining interval histories and physicals, collecting and routing blood samples, producing laboratory requisition slips, specimen labeling, and initial screening of complaints that might constitute adverse reactions, are the responsibility of the Center for Vaccine Development and/or its designee.

7.3 Investigational Supplies

Investigational supplies will be prepared and stored at Apovia, Inc. GMP manufacturing facility in Coralville, Iowa. All investigational supplies that are released will be distributed through the DMID Repository. The product will be transported under controlled temperature conditions.

7.3.1 Investigational Product Description

ICC-1132 with alhydrogel: This will be in 2 ml glass vials containing ICC-1132 at either 40 μg/ml or 100 μg/ml concentration formulated with alhydrogel at 1 mg/ml, with flip-off tops and butyl septa. Each vial will contain approximately 0.8 ml solution to permit recovery of 0.5 ml for injection. When shaken, the solution is an off-white to greyish-white turbid liquid free of foreign particulate matter.

7.3.2 Documentation of Vaccine Condition on Arrival at Study Site

Upon receipt of the vaccine shipment by the Investigational Drug Service (IDS) in Baltimore, the vaccine will be visually inspected.

7.3.3 Storage Requirements

ICC-1132 in alhydrogel: Store at 2–8 °C

7.3.4 Vaccine Accountability

At the termination of the study or at the request of the IND Sponsor, all used and unused vials of study drug will be returned to the DMID Repository. This return will be documented on the Clinical Product Tracking form supplied by Apovia, Inc.

7.4 Study Methods

Each subject will undergo the following procedures on the days indicated. All visits and procedures will take place at the CVD outpatient unit in Baltimore or at the GCRC unit at the University of Maryland Medical System hospital in Baltimore.

7.4.1 Recruitment

Recruitment will take place at the CVD outpatient unit in Baltimore. Typically adds are placed in local papers and flyers are posted, occasional radio advertisements may be used and the study may be advertised on the world wide web. Interested persons call the CVD for further information. They are asked to come in for screening and further explanation if they are interested and appear to meet inclusion and exclusion criteria.

7.4.2 Screening and Enrollment Process

The following activities take place during the screening process. Please see Section 5 on page 20 for further details about the consent process for *Screening* and *Enrollment*. They are listed below.

- Explain the HIPAA Consent forms and have the volunteer sign the forms.
- Explain the Screening Consent form and have the volunteer sign the form.
- Explain the HIV Informed Consent and obtain a signed copy from the volunteer.
- Complete medical history (including menstrual and contraceptive history and/or history of surgical sterility for female subjects).
- Administer physical examination (vital signs and basic physical exam emphasizing examination of any acute complaints).
- Obtain approximately 25 ml of blood for hematology (5 ml), biochemistry (5 ml) and serology tests (15 ml).
- Abnormal screening laboratory values will be repeated one time, and if normal, the repeat value will be used for screening purposes.
- For female volunteers only, obtain 2 ml serum for β-hCG test. Only females with negative serum β-hCG test results will be allowed in the study. Instruct all female volunteers to avoid becoming pregnant during the study.
- Obtain urine sample for urinalysis.
- Explain the study and Research Consent to the potential volunteer. Give a copy of the Research Consent document to the volunteer to take home for detailed review.

A subject will be considered *eligible for enrollment* when all the above procedures are complete. Eligible subjects may be asked to return to the clinic, if necessary, to complete the enrollment process. The following will be done:

- Review the Research Consent Form and the study with the volunteer. This may take place on an individual basis or as a group (see Section 5 on page 20).
- Administer the written exam.
- Grade the written exam, and review the results with the volunteer. In the event that the volunteer has not received a score $\geq$ 70%, the study coordinator or an Investigator will review the study and the Informed Consent with the volunteer. A volunteer who does not receive a score of $\geq$70% will be allowed to retake the exam one time before being considered ineligible for study enrollment.
- Ensure the subject has passed the Research Consent written exam.
- Subject and PI representative will sign the Research Consent form.
- Subject receives a signed copy of the Research Consent form.

A subject will be considered *eligible for vaccination* when all the above procedures are complete, all screening lab tests have been determined to be acceptable and the subject and PI representative have signed the Research Consent form.

7.4.3 Timing of Screening Labs

Screening tests will be performed within 28 working days of vaccination. If the screening took place more than seven days before the first vaccination, values for CBC (WBC and differential, platelet count, Hgb), creatinine, AST, and ALT shall be repeated within the seven days before the first vaccination.

7.4.4 Contraindications to Vaccination

The following criteria will be checked prior to each study vaccine injection and are contraindications to further study vaccine injections. However, the study participants will be encouraged to remain in the safety evaluation for the duration of the study.

- Hypersensitivity reaction following administration of the study vaccine (applies to injections number two and three only)
- Positive urine β-hCG

7.4.5 Indications for Deferral of Vaccination

The following adverse events constitute grounds for deferral of vaccine administration at that point in time; if any one of these adverse events occurs at the time scheduled for vaccination, the subject may be vaccinated at a later date, within the time interval specified in the protocol (Section 7.1 on page 24), or withdrawn at the discretion of the investigators. The subject must be followed until resolution of the event, or until non-causality is assigned to the event, as with any adverse event. A subject who is withdrawn from the study will be encouraged to remain in the safety evaluation for the duration of the study.

- Oral temperature $>$ 37.5 °C, with or without localizing or general symptoms, at the time of vaccination will warrant deferral of immunization until fever and symptoms resolve.

- Any other condition, that in the opinion of the investigator, poses a threat to the individual if immunized or that may complicate interpretation of the safety of the vaccine following immunization.

Such individuals will be followed daily until the symptoms resolve or the window for immunization expires. If the individual meets any of the above criteria for deferral on the day of first study vaccine injection, the PI may elect to exclude the subject from further participation in the study.

7.4.6 Detailed Description of Study Visits

The study events calendar is detailed in Section 12 on page 47. Screening events are described in Section 7.4.2 on page 27. Events for individual study days are presented in detail below.

7.4.6.1 Study Day 0 (Baseline, Day of First Injection)

1. Perform interim history and a basic physical exam emphasizing examination of any acute complaints.
2. Obtain approximately 105 ml of blood for hematology (5 ml), biochemistry (5 ml), serology tests (15 ml), CMI (75 ml) and antibody avidity assays (5 ml).
3. For female volunteers, obtain a urine sample for β-hCG test. Females will not undergo vaccination until the test is completed and is reported as negative.
4. Record vital signs (temperature, heart rate, blood pressure and respiratory rate).
5. Administer study vaccine and evaluate for adverse events for 30 minutes after injection. Evaluation will include an arm check, which will be documented.
6. Upon discharge from clinic, each volunteer will receive the event diary and an information sheet that provides a list of symptoms to watch for and information how to contact the Study Physician in the event of problems.

7.4.6.2 Study Day 1 (24 $\pm$ 6 hours after First Injection)

1. Perform interim history.
2. Perform arm check.
3. Record vital signs (temperature, heart rate, blood pressure and respiratory rate).

7.4.6.3 Study Day 2 (48 $\pm$ 6 hours after First Injection)

1. Perform interim history.
2. Perform arm check.
3. Record vital signs (temperature, heart rate, blood pressure and respiratory rate).
4. Obtain approximately 10 ml of blood for hematology (5 ml) and biochemistry (5 ml) tests.

7.4.6.4 Study Day 4 $\pm$ 1

1. Study nurse will perform safety interview by telephone. Questions will ascertain the presence or absence of symptoms contained in the daily symptom diary (see Section 7.4.7 on page 33). Symptoms will be graded according to the scale in the symptom diary.

7.4.6.5 Study Day 7 ± 1

1. Perform interim history and a basic physical exam emphasizing examination of any acute complaints.
2. Perform arm check.
3. Record vital signs (temperature, heart rate, blood pressure and respiratory rate).
4. Collect diary.

7.4.6.6 Study Day 14 ± 2

1. Perform interim history.
2. Record vital signs (temperature, heart rate, blood pressure and respiratory rate).
3. Obtain approximately 105 ml of blood for hematology (5 ml), biochemistry (5 ml), serology tests (15 ml), CMI (75 ml) and antibody avidity assays (5 ml).

7.4.6.7 Study Day 28 ± 4

1. Perform interim history.
2. Obtain approximately 95 ml of blood for serology (15 ml), CMI (75 ml) and antibody avidity assays (5 ml).

7.4.6.8 Study Day 56 ± 4 (Day of Second Injection)

1. Perform interim history and a basic physical exam emphasizing examination of any acute complaints.
2. Obtain approximately 105 ml of blood for hematology (5 ml), biochemistry (5 ml), serology tests (15 ml), CMI (75 ml) and antibody avidity assays (5 ml).
3. For female volunteers, obtain a urine sample for β-hCG test. Females will not undergo vaccination until the test is completed and is reported as negative.
4. Record vital signs (temperature, heart rate, blood pressure and respiratory rate).
5. Confirm that volunteer meets inclusion criteria, no exclusion criteria are met and there is no individual or cohort contraindication to further study vaccine injections.
6. Administer vaccine and evaluate for adverse events for 30 minutes after injection.
7. Each volunteer will receive a daily symptom diary in which to record symptoms and signs following vaccination.

7.4.6.9 Study Day 57 ± 4 (24 ± 6 hours after Second Injection)

1. Perform interim history.
2. Perform arm check.
3. Record vital signs (temperature, heart rate, blood pressure and respiratory rate).

7.4.6.10 Study Day 58 ± 4 (48 ± 6 hours after Second Injection)

1. Perform interim history.
2. Perform arm check.
3. Record vital signs (temperature, heart rate, blood pressure and respiratory rate).
4. Obtain approximately 10 ml of blood for hematology (5 ml) and biochemistry (5 ml) tests.

7.4.6.11 Study Day 60 ± 4 (4 ± 1 days after Second Injection)

1. Study nurse will perform safety interview by telephone. Questions will ascertain the presence or absence of symptoms contained in the daily symptom diary (see Section 7.4.7 on page 33). Symptoms will be graded according to the scale in the symptom diary.

7.4.6.12 Study Day 63 ± 5 (7 ± 1 days after Second Injection)

1. Perform interim history and a basic physical exam emphasizing examination of any acute complaints.
2. Perform arm check.
3. Record vital signs (temperature, heart rate, blood pressure and respiratory rate).
4. Collect diary.

7.4.6.13 Study Day 70 ± 6 (14 ± 2 days after Second Injection)

1. Perform interim history.
2. Record vital signs (temperature, heart rate, blood pressure and respiratory rate).
3. Obtain approximately 105 ml of blood for hematology (5 ml), biochemistry (5 ml), serology tests (15 ml), CMI (75 ml) and antibody avidity assays (5 ml).

7.4.6.14 Study Day 84 ± 8 (28 ± 4 days after Second Injection)

1. Perform interim history.
2. Obtain approximately 95 ml of blood for serology (15 ml), CMI (75 ml) and antibody avidity assays (5 ml).

7.4.6.15 Study Day 140 ± 11 (84 ± 7 days after Second Injection)

1. Obtain approximately 95 ml of blood for serology (15 ml), CMI (75 ml) and antibody avidity assays (5 ml).

7.4.6.16 Study Day 168 ± 14 (Day of Third Injection)

1. Perform interim history and a basic physical exam emphasizing examination of any acute complaints.
2. Obtain approximately 105 ml of blood for hematology (5 ml), biochemistry (5 ml), serology tests (15 ml), CMI (75 ml) and antibody avidity assays (5 ml).
3. For female volunteers, obtain a urine sample for β-hCG test. Females will not undergo vaccination until the test is completed and is reported as negative.
4. Record vital signs (temperature, heart rate, blood pressure and respiratory rate).
5. Confirm that volunteer meets inclusion criteria, no exclusion criteria are met and there is no individual or cohort contraindication to further study vaccine injections.
6. Administer vaccine and evaluate for adverse events for 30 minutes after injection.
7. Each volunteer will receive a daily symptom diary in which to record symptoms and signs following vaccination.

7.4.6.17 Study Day 169 ± 14 (24 ± 6 hours after the Third Injection)

1. Perform interim history.
2. Perform arm check.
3. Record vital signs (temperature, heart rate, blood pressure and respiratory rate).

7.4.6.18 Study Day 170 ± 14 (48 ± 6 hours after Third Injection)

1. Perform interim history.
2. Perform arm check.
3. Record vital signs (temperature, heart rate, blood pressure and respiratory rate).
4. Obtain approximately 10 ml of blood for hematology (5 ml) and biochemistry (5 ml) tests.

7.4.6.19 Study Day 172 ± 15 (4 ± 1 days after Third Injection)

1. Study nurse will perform safety interview by telephone. Questions will ascertain the presence or absence of symptoms contained in the daily symptom diary (see Section 7.4.7 on the following page). Symptoms will be graded according to the scale in the symptom diary.

7.4.6.20 Study Day 175 ± 15 (7 ± 1 days after Third Injection)

1. Perform interim history and a basic physical exam emphasizing examination of any acute complaints.
2. Perform arm check.
3. Record vital signs (temperature, heart rate, blood pressure and respiratory rate).
4. Collect diary.

7.4.6.21 Study Day 182 ± 16 (14 ± 2 days after Third Injection)

1. Perform interim history.
2. Record vital signs (temperature, heart rate, blood pressure and respiratory rate).
3. Obtain approximately 105 ml of blood for hematology (5 ml), biochemistry (5 ml), serology tests (15 ml), CMI (75 ml) and antibody avidity assays (5 ml).

7.4.6.22 Study Day 196 ± 18 (28 ± 4 days after Third Injection)

1. Perform interim history.
2. Obtain approximately 95 ml of blood for serology (15 ml), CMI (75 ml) and antibody avidity assays (5 ml).

7.4.6.23 Study Day 224 ± 21 (56 ± 7 days after Third Injection)

1. Obtain approximately 105 ml of blood for serology (15 ml), CMI (75 ml), study close screening serology (10 ml) and antibody avidity assays (5 ml).

7.4.6.24 Study Day 336 ± 28 (168 ± 14 days after Third Injection)

1. Study nurse will perform telephone interview for long-term safety follow-up. The aim of this interview is to elicit the presence of any new adverse events or chronic illnesses that have occurred since the final immunization.
2. Optionally, the volunteer will be present in person to answer the same follow-up questions as would be required during the telephone call, but additionally they will give approximately 95 ml of blood for serology (15 ml), CMI (75 ml) and antibody avidity assays (5 ml).

7.4.7 Volunteer Symptom Diary

For seven days after receiving each injection of vaccine, volunteers will keep a daily symptom diary. The absence or presence of the following symptoms will be ascertained: subjective fever, chills, headache, rash, anorexia, nausea, vomiting (if present, number of episodes will be recorded), stomach ache, muscle aches, joint aches, photophobia, itching, and pain at injection site. Symptoms will be graded as follows:

0	Symptom absent
1	Mild; no change in activity and/or no medication necessary
2	Moderate; requires change in activity and/or medication
3	Severe; bed rest required and/or medical intervention other than medication alone (such as outpatient visit in emergency department or clinic, excludes hospitalization)

The diary will have extra space for additional comments volunteers may wish to add.

Volunteers will also be asked to take an oral temperature reading upon waking up and another one at approximately 8:00 PM each evening. The result of each measurement will be recorded on the symptom diary. Oral thermometers will be provided for these measurements. There may be no recorded AM temperature on the day of vaccination because volunteers have their temperatures measured and recorded by study staff at that time.

7.4.8 Laboratory Testing

Using standard techniques LabCorp will perform the following screening tests: WBC with differential, platelets, hemoglobin, creatinine, AST, ALT, serum glucose, hepatitis C antibody, hepatitis B surface antigen, hepatitis B surface antibody, hepatitis B core antibody, HIV serology, serum β-hCG (on women only), and urinalysis.

Follow-up laboratory work to monitor each vaccination will include serum creatinine, ALT, AST, hemoglobin, platelets, WBC and differential count.

Urine β-hCG test will be performed before each vaccination using an FDA approved urine pregnancy test kit.

Assays of humoral and cellular immunity will be carried out in the Department of Medical and Molecular Parasitology, New York University School of Medicine (see Section 7.6 for details). Additional antibody assays to measure anti-hepatitis B titers and antibody avidity will be performed at Apovia, Inc. San Diego, California.

7.5 Volunteer Compensation

Volunteers will be paid a stipend of $100 for each vaccination day (n=3), $30 for each outpatient visit to donate specimens, undergo clinical evaluation or illicit history (n=18), which includes possible extra screening blood specimens, $49 for completion of each symptom diary (n=3), $10 for each phone follow-up (n=4) and $100 for successful completion of the study. Estimated total compensation will be at least $1,127 per volunteer completing the study.

7.6 Immunologic Assays

The results of the immunologic assays will be used to determine the dosage of vaccine that will be given in future studies. The primary outcome for dosage selection will be the antibody response. As a general guide, IFA titers greater than 1:5120 will be considered an indicator of immunity for any given cohort. This IFA titer is based upon results of an earlier CSP vaccine trial in which six of seven volunteers with high anti-CSP titers resisted experimental malaria (25).

The protocols for the laboratory assays are summarized below (39;41;44;45).

7.6.1 Serologic Assays

Serum from each volunteer, obtained at the time of each immunization and at 14 and 28 days after each immunization, will be tested for anti-sporozoite and anti-hepatitis B core antibodies. Persistence of vaccine-induced humoral responses will also be assayed in sera samples collected at day 84 following the second vaccination, at day 56 after the third vaccination and possibly at day 168 after the third vaccination.

The titer of antibody specific for the *P. falciparum* malaria epitopes will be determined by peptide-based ELISA. Reactivity with CSP repeat or non-repeat epitopes will be measured using MAPs containing repeats $(T1, B)_4$ or the universal T cell epitope (T*), respectively (41;44;45). To better characterize the serological responses in the vaccinees, antibody isotype and IgG subclasses will be determined in sera with high ELISA titers.

Sporozoite-specific IgG antibodies will be measured by indirect immunofluorescent antibody assays (IFA) using glutaraldehyde-fixed or air-dried *P. falciparum* sporozoites (41;45). Based on ELISA and/or IFA results, selected sera samples will be tested for reactivity with viable *P. falciparum* sporozoites using the circumsporozoite precipitin assay (41;45). For this assay, viable sporozoites are mixed with two-fold dilutions of serum and incubated at 37 °C for 40 minutes. A total of twenty parasites in each sample are examined by phase microscopy for the presence of the characteristic terminal circumsporozoite precipitin. Positive sera

are defined as samples that elicit circumsporozoite precipitin reactions on a minimum of ten percent of *P. falciparum* sporozoites.

IgG subtypes (IgG1, IgG2, IgG3 and IgG4) and IgE antibodies will be determined in selected sera.

Antibodies to the hepatitis B virus core antigen will also be measured using commercial and laboratory ELISA. Quantification of anti-hepatitis B virus core antibody levels will be obtained using a commercial kit. ELISA will also be carried out using laboratory protocols to measure antibodies specific for immunogen, or for core particle alone, using antigens produced by Apovia.

7.6.1.1 Antibody Avidity Assays The avidity of the antibodies specific for the B-cell epitope $(\text{NANP})_3$ will be investigated using an ELISA (46;47). This assay has not been validated. A brief description of the assay follows.

Microtiter plates are coated with $(\text{NANP})_5$ peptide and probed with various dilutions of sera in the presence of increasing concentrations (from 0 to 6 M) of the chaotropic agent potassium thiocyanate. The higher the concentration of thiocyanate ions required to inhibit binding, the greater the avidity of the antibodies.

7.6.2 Cellular Assays for Cell-Mediated Immunity (CMI)

Peripheral blood lymphocytes (PBL) of each volunteer will be obtained at the time of immunization and at 14 and 28 days post each immunization. Kinetics and persistence of cellular responses will be determined using samples collected at day 84 following the second vaccination and at day 56 after the third vaccination. Ficoll purified PBL will be tested for malaria-specific and hepatitis-specific cellular responses using proliferation and cytokine assays (41;45).

In the proliferation assay, PBL will be incubated in triplicate wells with various concentrations of CSP-HBcAg, hepatitis B core alone, or linear peptides representing the repeats $(\text{T1, B})_4$ or universal T cell epitope (T*). Culture wells tritiated with ^{3}H-Tdr on day five will be incubated overnight and harvested. The results will be expressed as stimulation index (SI). Cytokines will be measured using culture supernatants incubated for two days or five days with antigen. IL-2 will be detected using an IL-2 dependent cell line bioassay and the results expressed as SI. IL-4 and/or IL-10 levels will be measured in selected cell supernatants using commercial cytokine kits (R&D Systems, Inc., Minneapolis, MN).

Gamma interferon (γ-IFN) production by PBL, expanded six days in vitro with rCSP or core antigen, will be measured using a commercial enzyme-linked immunospot (ELISPOT) kit (BD PharMingen, San Diego, CA). The cells will be incubated overnight with malaria peptides, or hepatitis core antigen, in wells coated with antibody specific for human γ-IFN. After washing and addition of biotinylated anti-IFN, followed by alkaline phosphatase-conjugated streptavidin, capture of γ-IFN can be visualized by reaction with the chromogenic enzyme substrate BCIP/NBT. Individual cells producing γ-IFN will be enumerated by counting the colored spots formed by the precipitated substrate and the results expressed as spot forming cells (SFC)/10^6 cells.

Peripheral blood lymphocytes obtained from vaccinees at each time point will also be expanded with rCSP in order to establish T cell lines (TCL). The TCL will be tested for proliferation using peptide-pulsed autologous Epstein-Barr virus-transformed B cells (EBV-B) as antigen presenting cells. Cytokines will be measured in culture supernatants using the IL-2 bioassay and IL-4 or IL-10 ELISA (R&D Systems, Inc., Minneapolis, MN). Interferon production will be measured by ELISPOT, as described for PBL.

8 Adverse Events

All adverse events occurring in participants after administration of vaccine will be reported as described below, in compliance with 21 CFR 312.32.

8.1 Definitions

Adverse event (AE): An AE is any untoward medical occurrence, including a dosing error, that may present during vaccination with ICC-1132 or after administration of ICC-1132.

The AE may or may not have a causal relationship with vaccination as indicated by physical signs, symptoms, and/or clinically significant laboratory abnormalities that occur. The definition includes intercurrent illnesses, injuries, exacerbation of pre-existing conditions, and events occurring as a result of product misuse or overdose.

A change in a *laboratory variable* is considered an adverse event if it:

- leads to a change in the subject's functional status
- is considered by the attending physician to be clinically significant, or
- if it caused the clinician to reduce or discontinue the use of the product, or institute a specific therapy.

8.2 Adverse Events Assessment

Every adverse event will be recorded on the Case Report Form and will include a causal assessment by the Investigator. All AE's of greater than grade 1 will be recorded in the AE Log. All adverse events will be followed until resolution of the symptom or laboratory change occurs, or until a non-study related causality is assigned.

8.2.1 Assessment of Intensity

Adverse events will be graded using criteria from the Division of Microbiology and Infectious Diseases adult toxicity table (May 2001) as defined in Section 14 on page 52, unless stated otherwise in the protocol.

8.2.2 Assessment of Causality

For each adverse event, the investigator will assess the causal relationship, if any, to administration of the study vaccine. An intervention-related adverse event refers to an adverse event for which there is a probable or definite relationship to administration of vaccine (ICC-1132 formulated with alhydrogel). The Principle Investigator will interpret the causal relationship of the intervention to the adverse event in question. This interpretation will be based on the type of event, the relationship of the event to the time of vaccine administration, and the known biology of the vaccine therapy. The following are guidelines for assessing the relationship of ICC-1132 administration to the AE.

1. Unrelated
   - No temporal relationship to study product
   - Alternate etiology (clinical state, environmental or other interventions) established
   - Does not follow known pattern of response to study product
   - Does not reappear or worsen with re-challenge
2. Unlikely or Remote
   - No temporal relationship to study product
   - Event could readily be produced by clinical state, environmental or other interventions

- Does not follow known pattern of response to study product
- Does not reappear or worsen with re-challenge

3. Possible Relationship
   - Reasonable temporal relationship to study product
   - Event not readily produced by clinical state, environmental or other interventions
   - Known pattern of response to study product or unknown pattern of response to study product with a newly defined safety profile

4. Probable Relationship
   - Reasonable temporal relationship to study product
   - Event not readily produced by clinical state, environment or other interventions
   - Known pattern of response to study product

5. Definite Relationship
   - Reasonable temporal relationship to study product
   - Event not readily produced by clinical state, environment or other interventions
   - Known pattern of response to study product
   - Recurs with re-challenge

8.2.3 Pregnancy

Participants who become pregnant during the study period must not receive additional doses of vaccine, but will be encouraged to continue other study procedures. Female participants will be instructed to notify the investigators if they become pregnant at any time during the 12-month study period. Although not considered an adverse event, pregnancy will be reported in the same way as a Serious Adverse Event. All pregnancies occurring during the study period will be followed to term, any premature termination reported, and the health status of the mother and child, including date of delivery and the child's gender and weight, will be reported to the SMC who will be responsible for informing the DMID.

8.2.4 Treatment of Adverse Events

Treatment of any adverse event will be provided by the Investigators with advice from the Local Medical Monitor and according to current Good Medical Practice. The applied measures will be recorded in the CRF of the participant. The recording of adverse events is an important aspect of study documentation. It is the responsibility of the Investigators to document all adverse events according to the detailed guidelines set out in this protocol. The participants will be instructed to contact the investigators immediately if they manifest any signs or symptoms they perceive as serious.

8.3 Serious Adverse Events

8.3.1 Definitions of Serious Adverse Events

Serious adverse event (SAE): Any adverse event (whether considered related to the investigational product or not) that results in any of the following outcomes:

Death (i.e., results in death from any cause at any time)

Life-threatening event (i.e., the subject was, in the view of the investigator, at immediate risk of death from the event that occurred) This does not include an adverse event that, if it occurred in a more serious form, might have caused death.

Persistent or significant disability or incapacity (i.e., substantial disruption of one's ability to carry out normal life functions)

Hospitalization An overnight stay in the hospital, regardless of length of stay, even if the hospitalization is a precautionary measure for continued observation. Hospitalization (including inpatient or outpatient hospitalization for an elective procedure) for a preexisting condition that has not worsened unexpectedly, does not constitute a serious adverse event.

Important medical event (that may not cause death, be life threatening, or require hospitalization) that may, based upon appropriate medical judgment, jeopardize the subject and/or require medical or surgical intervention to prevent one of the outcomes listed above. Examples of such medical events include allergic reaction requiring intensive treatment in an emergency room or clinic, hematologic abnormalities or convulsions that do not result in inpatient hospitalization.

Congenital anomaly or birth defect

8.3.2 Reporting

All serious adverse events will be reported by telephone, email or FAX to the University of Maryland at Baltimore IRB and the NIAID within one working day of notification of SAE occurrence. At the NIAID, the report will be sent via FAX to both Holli Hamilton (301-435-3649) and B. Fenton Hall (301-402-0659). Holli Hamilton will be responsible for forwarding the report to the Regulatory Affairs group at the DMID. The IND Sponsor will be responsible for reporting the adverse events to the FDA.

8.4 Adverse Events Monitoring

For this study, an Independent Safety Monitor (ISM) was appointed to provide real-time safety oversight. The ISM, or their appointee, will review serious adverse events immediately after they occur and follow these events until resolution.

A Safety Monitoring Committee (SMC) appointed by DMID will undertake regular review of the study. The SMC will review all adverse events. The SMC will evaluate individual and cumulative participant data in formulating recommendations regarding the safe continuation of the study.

8.4.1 Role of Independent Safety Monitor

Dr. Carol Tacket is the ISM for this study. The ISM is a physician with relevant expertise whose primary responsibility is to provide independent safety monitoring in a timely fashion. The ISM is responsible for ongoing, regularly occurring review of safety data. The ISM's responsibilities will include:

- review of serious adverse events within 24 hours of their receipt

- review of the occurrence of severe solicited and unsolicited abnormalities during the fourteen day period following each vaccination to determine the feasibility of dose escalation
- timely review of adverse events that are reported at other times during the study

The ISM will confer with the Safety Monitoring Committee before continuing with dose escalation or resumption of an individual or cohort's entry into the study. In addition, the ISM will confer with the Safety Monitoring Committee immediately regarding any safety issues that might result in dose interruption for an individual volunteer or cohort.

The ISM, or their appointee, will clinically assess all volunteers with grade 3 or 4 reactions, and volunteers with grade 2 reactions, if persistent in the judgment of the investigators. If an appointee of the ISM is used to evaluate volunteers, the ISM and their appointee must document that they have discussed the case and enter this documentation into the volunteer's research record.

8.4.1.1 ISM Criteria to Suspend the Trial If there is concern for the safety of any volunteer at any time including, but not limited to those events bulleted above (Section 8.4.1 on the page before), the ISM may convene a meeting of the SMC. The ISM *must* convene a meeting of the SMC prior to suspending the trial or suspending further vaccinations for any cohort or individual.

The ISM has the power to suspend dose escalation and/or additional injections in all study cohorts and/or individuals, pending final review by the SMC, if any of the following occurs:

- One or more subjects experiences a serious adverse reaction (as defined in Section 8.3.1 on the preceding page)
- One or more subjects experiences anaphylaxis
- Any severe clinical illness not explained by a diagnosis unrelated to vaccination with ICC-1132

8.4.2 Role of Safety Monitoring Committee

A Safety Monitoring Committee (SMC), appointed by the DMID, will provide safety oversight by review of individual and cumulative participant data. The primary responsibilities of the SMC are to 1) periodically review and evaluate the accumulated study data for participant safety, study conduct, and study progress, and 2) make recommendations to the DMID concerning the continuation, modification, or termination of the trial. The SMC will meet regularly and whenever any special need arises to review study conduct and cumulative study data, and to recommend whether the study should continue without change, be modified, or be terminated. The SMC will review safety data and all decisions made by the Independent Safety Monitor that deal with the interruption or resumption of entry into the study by any cohort or individual volunteer. The SMC may also recommend that an individual volunteer(s) be discontinued from the trial. Recommendations to modify, suspend or terminate the trial may be based on any aspects of the trial it considers.

8.4.2.1 Analysis Plan for the SMC The analysis plan for the SMC will be qualitative in nature and will involve review of the volunteer-reported symptoms from the seven day Symptom Diary, any additional unsolicited symptoms, findings from physical examination, and any changes in laboratory values from baseline to day 14. These events will be presented in a tabular form to the SMC. The proportion of volunteers in a cohort experiencing a given event and the severity of this event will be calculated. The SMC will review data from each dose cohort sequentially.

Safety analysis will be done on four separate levels: the individual volunteer, the study cohort (ICC-1132 in saline or ICC-1132 + alhydrogel (10 μg cohort only)), the study arm (10, 20 or 50 μg dose cohorts) and the entire trial.

8.4.2.2 SMC Criteria to Suspend the Trial The SMC will be responsible for determining whether a dose of vaccine should be considered unacceptably reactogenic as defined by the following criteria:

- One or more subjects experiences a serious adverse reaction (as defined in Section 8.3.1 on page 38)
- One or more subjects experiences anaphylaxis
- Any severe clinical illness not explained by a diagnosis unrelated to vaccination with ICC-1132
- Two or more subjects in a single dose cohort experience an objective physical finding, symptom or laboratory abnormality of grade 3 or 4 that is of possible, probable or definite relationship to vaccination

Evaluation of reactogenicity will be done separately for each dosage cohort within each study arm (e.g. the 10 μg dose in the ICC-1132 in saline arm, the 10 μg dose in the ICC-1132 + alhydrogel arm) for the 10 μg dose cohort only.

Following the day 14 visit, adverse events and laboratory data from day 0 through day 14 post-injection will be compiled in a tabular form, as well as a brief narrative summary. This table will include a summary of volunteer reported symptoms (including the symptom diary data from days zero through six), findings on physical examination, and laboratory test results. All symptoms, physical findings, and laboratory results will be graded (see Section 14 on page 52). If a symptom, sign, or lab result occurs in an individual volunteer more than once, the highest grading will be reported. The 14-day summary will be done for each dose of the vaccine.

The Independent Safety Monitor and the Safety Monitoring Committee will assess the clinical significance of any adverse events occurring during the 14-day period following vaccination and prior to dose escalation. As an example, mild or moderate solicited adverse events (which are expected) would be unlikely to preclude further dose escalation. In contrast, severe solicited events, or moderate or severe unsolicited events, such as a severe migraine headache in a volunteer with no prior history of migraines, would require further evaluation and may preclude further dose escalation.

The summary data set will be sent to the NIAID Program Officer. The Program Officer will then forward this information to members of the SMC. A conference call will be arranged by the DMID and will include at a minimum the Principal Investigator, the ISM, the SMC and the Program Officer. Any and all adverse events will be reviewed during this conference call. After this review, the Principal Investigator and the Program Officer will be excused from the call. The remaining participants will make recommendations regarding the safe continuation of the study. During the closed session only voting members of the SMC will make recommendations.

Based on its deliberations, the SMC may:

1. Recommend to continue the study as planned
2. Recommend to prohibit further vaccination of one or more individuals
3. Recommend to discontinue participation of all individuals in a particular dose group or arm
4. Recommend to discontinue the trial; or
5. Make other recommendations regarding the conduct of the trial.

An independent recorder will be present during the discussion and draft a summary of the recommendations. The Chair of the SMC will communicate to the NIAID the results of the deliberations.

After each review, the SMC will provide verbal and written reports to the DMID Program Officer. These reports will be forwarded to the Principal Investigator. Findings of a serious and immediate nature including

any recommendations to discontinue all or part of the trial must be reported in verbal and written forms to the DMID Program Officer immediately.

In the event that an individual, a cohort, an arm or the study is discontinued, participants will continue to be monitored for purposes of safety according to the original study schedule.

8.5 Criteria for Study Termination

- The SMC believes the trial should be terminated based upon their independent review of the accumulated study data
- The SMC deems the vaccine unacceptably reactogenic (as defined in Section 8.4.2.2 on the page before)
- The Investigators believe that continuing the protocol may be deleterious to the health of study participants

9 Analysis

9.1 Expected Outcome

This study is expected to provide a preliminary assessment of the safety of ICC-1132 + alhydrogel in healthy adults and to determine if there are any probable or definitive SAEs. The study is also expected to provide valuable insight into the immunogenicity of ICC-1132 in saline and ICC-1132 + alhydrogel in healthy human subjects, though immunogenicity data for the saline formulation will not be as complete as hoped because of the discontinuation of this formulation after only 17 doses.

9.2 Sample Size Calculation

Because this is a descriptive study focusing on safety and immunogenicity, sample sizes were derived from logistic considerations, rather than by power analyses. The maximum number of enrolled volunteers has been increased to reflect the 38% drop-out rate, the majority before receiving their first vaccination. We will enroll a maximum of 80 individuals.

9.3 Randomization Procedure

Subjects in the 10 μg cohort and the first three subjects in the 20 μg cohort were randomized to receive ICC-1132 + saline or ICC-1132 + alhydrogel by computer randomization. Specifically, using SAS, a randomization list was prepared in advance of vaccination activities with randomized blocks of N = 2 (one subject receiving saline and the other alhydrogel). Assignments to vaccine groups from this randomization were placed in individual sealed envelopes and were provided to the immunization team. With the removal of the saline formulated vaccine all subsequent subjects will receive ICC-1132 + alhydrogel, so there is no need for continued randomization.

Subjects will be assigned personal identification numbers (PIDs) in the order of their arrival for their first immunization (note that PIDs are not issued at the time of enrollment, but just prior to vaccination).

9.4 Safety Analysis

A safety profile of the 10 μg cohort of the ICC-1132 in saline (eight volunteers) and ICC-1132 adjuvanted with alhydrogel (eight volunteers) will be compiled from analysis of historical, physical, and laboratory information. The safety profile of the 20 μg and 50 μg alhydrogel groups will not be blinded.

Using each subject's seven-day symptom diary (three diaries per volunteer), clinical exam (arm check) and laboratory data, the presence of symptoms/signs and the peak severity of graded measures will be calculated. The point estimate of incidence of each symptom/sign occurring at any time over the seven-day period (i.e., number of subjects positive/total number of subjects) will be calculated for each study arm. Similarly, the frequency distribution of severity for each graded sign/symptom during the seven-day period will be calculated. The Fisher's Exact Test (FET) for difference between vaccine + saline versus vaccine + adjuvant will be performed for the 10 μg dose cohort only. This will be evaluated at alpha = 0.05.

Early study termination should not terminate the collection of symptom diaries; hence, it is anticipated that complete diaries will be available for each immunization that is administered. If the study is terminated early, higher and/or lower dose groups may have incomplete safety data (if not all immunizations are administered). In such a case, only those groups and/or subjects with collected data will be included in the above analyses.

9.5 Immunologic Analysis

9.5.1 Serologic Responses

The main outcome variables to be analyzed include geometric mean IFA and ELISA antibody, percent with >4-fold IFA and ELISA antibody rise, and proportion with >1:5120 IFA and ELISA antibody.

Prior to their analysis, inverse antibody titers will be transformed to natural logarithms. The point estimate of the frequency of four-fold rises over baseline and of the proportion of subjects attaining >1:5120 antibody levels will be calculated for each assay. Geometric mean inverse titers also will be calculated. For dichotomous endpoints, analysis will proceed as presented above for safety endpoints. For dimensional endpoints, Wilcoxon's rank-sum test for difference between vaccine + saline vs. vaccine + adjuvant will be performed in the 10 μg dose cohort only. This will be evaluated at alpha = 0.05.

Results of these analyses will be interpreted in light of the association between consecutive immunological readouts. If the study is terminated early, higher and/or lower dose groups may have incomplete safety data (if not all immunizations are administered). In that case, only those groups and/or individuals with collected data will be included in the above analyses.

The number and proportion of volunteers in each vaccine dose cohort that seroconvert for hepatitis B core antibody will be calculated.

IgG subtypes (IgG1, IgG2, IgG3 and IgG4) and IgE antibodies will be determined in selected sera. The mean antibody concentration and range will be calculated. No inferential tests will be done.

9.5.1.1 Antibody Avidity Responses The antibody avidity will be calculated for each vaccine cohort at several study time points (see Table 2 on page 48 for details). Geometric mean and range of avidity, at each time point, for each group, will be calculated. Changes in avidity (peak/baseline) will also be calculated for each group. As a research question, we will compare vaccine in saline versus vaccine + adjuvant for the 10 μg dose and the portion of the 20 μg dose with both saline and alhydrogel formulations, by Wilcoxon's signed-ranks test. Each of the above to be evaluated at alpha = 0.05.

9.5.2 Cellular Assays of CMI Responses

In the proliferation assay, PBL will be incubated in triplicate wells with various concentrations of CSP-HBcAg, hepatitis B core alone, or linear peptides representing the repeats $(T1, B)_4$ or universal T cell epitope (T*). The results will be expressed as stimulation index (SI). The mean, SE, and median SI will be calculated, at each time point, for each group, within each vaccine cohort.

Gamma interferon production by PBL will be enumerated by counting the colored spots formed by the precipitated substrate and the results expressed as spot forming cells (SFC)/10^6 cells. The mean, SE, and median will be calculated at each time point, for each group, within each vaccine cohort.

IL-4, IL-2, and IL-10 will be determined in selected sera. The mean picograms per milliliter and range will be calculated selected time points in each group within each vaccine cohort. No inferential tests will be done.

10 Withdrawal from Study

Every reasonable effort should be made to maintain protocol compliance and participation in the study. Should a subject be prematurely terminated from the study for any reason, the reason for early study withdrawal will be recorded. If withdrawal is the result of a serious adverse event, the subject will be followed until the condition has resolved, as determined by the investigator. Subjects withdrawn prematurely for any reason will not be re-entered into this protocol. A complete evaluation should be completed for any subject who prematurely terminates from the study.

The following reasons will result in study termination and will not be considered normal protocol completion.

Developed an adverse event - applies to a subject who is withdrawn from the study primarily due to an adverse event, serious or otherwise.

Lost to follow-up - applies to a subject who does not return for protocol study visits, is not reachable by telephone or other means of communication and/or is not able to be located.

Research terminated by investigator - applies to the situation where the entire study or a portion of the study is terminated by the investigator for any reason.

Withdrawal of consent - applies to a subject who withdraws consent to participate in the study for any reason.

Non-compliant with protocol - applies to a subject who does not comply with protocol specific visits or evaluations even though the subject is able to comply.

Other- is a category used when previous categories do not apply and requires an explanation.

11 References

1. Greenwood BM, Bradley AK, Greenwood AM, Byass P, Jammeh K, Marsh K et al. Mortality and morbidity from malaria among children in a rural area of The Gambia, West Africa. Trans R Soc Trop Med Hyg 1987; 81(3):478-486.

2. Miller LH, Howard RJ, Carter R, Good MF, Nussenzweig V, Nussenzweig RS. Research toward malaria vaccines. Science 1986; 234(4782):1349-1356.

3. Berzofsky JA, Cease KB, Cornette JL, Spouge JL, Margalit H, Berkower IJ et al. Protein antigenic structures recognized by T cells: potential applications to vaccine design. Immunol Rev 1987; 98:9-52.

4. Schofield L, Villaquiran J, Ferreira A, Schellekens H, Nussenzweig R, Nussenzweig V. Gamma interferon, CD8+ T cells and antibodies required for immunity to malaria sporozoites. Nature 1987; 330(6149):664-666.

5. Nardin EH, Nussenzweig RS. T cell responses to pre-erythrocytic stages of malaria: role in protection and vaccine development against pre-erythrocytic stages. Annu Rev Immunol 1993; 11:687-727.

6. Nussenzweig V, Nussenzweig RS. Rationale for the development of an engineered sporozoite malaria vaccine. Adv Immunol 1989; 45:283-334.

7. Tsuji M, Romero P, Nussenzweig RS, Zavala F. CD4+ cytolytic T cell clone confers protection against murine malaria. J Exp Med 1990; 172(5):1353-1357.

8. Renia L, Marussig MS, Grillot D, Pied S, Corradin G, Miltgen F et al. In vitro activity of CD4+ and CD8+ T lymphocytes from mice immunized with a synthetic malaria peptide. Proc Natl Acad Sci U S A 1991; 88(18):7963-7967.

9. Moreno A, Clavijo P, Edelman R, Davis J, Sztein M, Herrington D et al. Cytotoxic CD4+ T cells from a sporozoite-immunized volunteer recognize the Plasmodium falciparum CS protein. Int Immunol 1991; 3(10):997-1003.

10. Clyde DF. Immunization of man against falciparum and vivax malaria by use of attenuated sporozoites. Am J Trop Med Hyg 1975; 24(3):397-401.

11. Rieckmann KH, Beaudoin RL, Cassells JS, Sell KW. Use of attenuated sporozoites in the immunization of human volunteers against falciparum malaria. Bull World Health Organ 1979; 57 Suppl 1:261-265.

12. Herrington D, Davis J, Nardin E, Beier M, Cortese J, Eddy H et al. Successful immunization of humans with irradiated malaria sporozoites: humoral and cellular responses of the protected individuals. Am J Trop Med Hyg 1991; 45(5):539-547.

13. Edelman R, Hoffman SL, Davis JR, Beier M, Sztein MB, Losonsky G et al. Long-term persistence of sterile immunity in a volunteer immunized with X-irradiated Plasmodium falciparum sporozoites. J Infect Dis 1993; 168(4):1066-1070.

14. Egan JE, Hoffman SL, Haynes JD, Sadoff JC, Schneider I, Grau GE et al. Humoral immune responses in volunteers immunized with irradiated Plasmodium falciparum sporozoites. Am J Trop Med Hyg 1993; 49(2):166-173.

15. Nussenzweig V, Nussenzweig RS. Circumsporozoite proteins of malaria parasites. Cell 1985; 42(2):401-403.

16. Zavala F, Tam JP, Hollingdale MR, Cochrane AH, Quakyi I, Nussenzweig RS et al. Rationale for development of a synthetic vaccine against Plasmodium falciparum malaria. Science 1985; 228(4706):1436-1440.

17. Young JF, Hockmeyer WT, Gross M, Ballou WR, Wirtz RA, Trosper JH et al. Expression of Plasmodium falciparum circumsporozoite proteins in Escherichia coli for potential use in a human malaria vaccine. Science 1985; 228(4702):958-962.

18. Ballou WR, Hoffman SL, Sherwood JA, Hollingdale MR, Neva FA, Hockmeyer WT et al. Safety and efficacy of a recombinant DNA Plasmodium falciparum sporozoite vaccine. Lancet 1987; 1(8545):1277-1281.

19. Herrington DA, Clyde DF, Losonsky G, Cortesia M, Murphy JR, Davis J et al. Safety and immunogenicity in man of a synthetic peptide malaria vaccine against Plasmodium falciparum sporozoites. Nature 1987; 328(6127):257-259.

20. Fries LF, Gordon DM, Schneider I, Beier JC, Long GW, Gross M et al. Safety, immunogenicity, and efficacy of a Plasmodium falciparum vaccine comprising a circumsporozoite protein repeat region peptide conjugated to Pseudomonas aeruginosa toxin A. Infect Immun 1992; 60(5):1834-1839.

21. Fries LF, Gordon DM, Richards RL, Egan JE, Hollingdale MR, Gross M et al. Liposomal malaria vaccine in humans: a safe and potent adjuvant strategy. Proc Natl Acad Sci U S A 1992; 89(1):358-362.

22. Hoffman SL, Edelman R, Bryan JP, Schneider I, Davis J, Sedegah M et al. Safety, immunogenicity, and efficacy of a malaria sporozoite vaccine administered with monophosphoryl lipid A, cell wall skeleton of mycobacteria, and squalane as adjuvant. Am J Trop Med Hyg 1994; 51(5):603-612.

23. Gordon DM, McGovern TW, Krzych U, Cohen JC, Schneider I, LaChance R et al. Safety, immunogenicity, and efficacy of a recombinantly produced Plasmodium falciparum circumsporozoite protein-hepatitis B surface antigen subunit vaccine. J Infect Dis 1995; 171(6):1576-1585.

24. Heppner DG, Gordon DM, Gross M, Wellde B, Leitner W, Krzych U et al. Safety, immunogenicity, and efficacy of Plasmodium falciparum repeatless circumsporozoite protein vaccine encapsulated in liposomes. J Infect Dis 1996; 174(2):361-366.

25. Stoute JA, Slaoui M, Heppner DG, Momin P, Kester KE, Desmons P et al. A preliminary evaluation of a recombinant circumsporozoite protein vaccine against Plasmodium falciparum malaria. RTS,S Malaria Vaccine Evaluation Group. N Engl J Med 1997; 336(2):86-91.

26. Stoute JA, Kester KE, Krzych U, Wellde BT, Hall T, White K et al. Long-term efficacy and immune responses following immunization with the RTS,S malaria vaccine. J Infect Dis 1998; 178(4):1139-1144.

27. Kester KE, McKinney DA, Tornieporth N, Ockenhouse CF, Heppner DG, Hall T et al. Efficacy of recombinant circumsporozoite protein vaccine regimens against experimental Plasmodium falciparum malaria. J Infect Dis 2001; 183(4):640-647.

28. Valero MV, Amador LR, Galindo C, Figueroa J, Bello MS, Murillo LA et al. Vaccination with SPf66, a chemically synthesised vaccine, against Plasmodium falciparum malaria in Colombia. Lancet 1993; 341(8847):705-710.

29. Alonso PL, Smith T, Schellenberg JR, Masanja H, Mwankusye S, Urassa H et al. Randomised trial of efficacy of SPf66 vaccine against Plasmodium falciparum malaria in children in southern Tanzania. Lancet 1994; 344(8931):1175-1181.

30. D'Alessandro U, Leach A, Drakeley CJ, Bennett S, Olaleye BO, Fegan GW et al. Efficacy trial of malaria vaccine SPf66 in Gambian infants. Lancet 1995; 346(8973):462-467.

31. Nosten F, Luxemburger C, Kyle DE, Ballou WR, Wittes J, Wah E et al. Randomised double-blind placebo-controlled trial of SPf66 malaria vaccine in children in northwestern Thailand. Shoklo SPf66 Malaria Vaccine Trial Group. Lancet 1996; 348 (9029):701-707.

32. Tam JP, Clavijo P, Lu YA, Nussenzweig V , Nussenzweig R, Zavala F. Incorporation of T and B epitopes of the circumsporozoite protein in a chemically defined synthetic vaccine against malaria. J Exp Med 1990; 171(1):299-306.

33. Zavala F, Chai S. Protective anti-sporozoite antibodies induced by a chemically defined synthetic vaccine. Immunol Lett 1990; 25(1-3):271-274.

34. Amador R, Moreno A, Murillo LA, Sierra O, Saavedra D, Rojas M et al. Safety and immunogenicity of the synthetic malaria vaccine SPf66 in a large field trial. J Infect Dis 1992; 166(1):139-144.

35. Teuscher T, Schellenberg JR, Bastos dA, I, Hurt N, Smith T, Hayes R et al. SPf66, a chemically synthesized subunit malaria vaccine, is safe and immunogenic in Tanzanians exposed to intense malaria transmission. Vaccine 1994; 12(4):328-336.

36. Noya O, Gabaldon BY, Alarcon dN, Borges R, Zerpa N, Urbaez JD et al. A population-based clinical trial with the SPf66 synthetic Plasmodium falciparum malaria vaccine in Venezuela. J Infect Dis 1994; 170(2):396-402.

37. Nosten F, Luxemburger C, Kyle DE, Gordon DM, Ballou WR, Sadoff JC et al. Phase I trial of the SPf66 malaria vaccine in a malaria-experienced population in Southeast Asia. Am J Trop Med Hyg 1997; 56(5):526-532.

38. Keitel WA, Kester KE, Atmar RL, White AC, Bond NH, Holland CA et al. Phase I trial of two recombinant vaccines containing the 19kd carboxy terminal fragment of Plasmodium falciparum merozoite surface protein 1 (msp-1(19)) and T helper epitopes of tetanus toxoid. Vaccine 1999; 18(5-6):531-539.

39. Moreno A, Clavijo P, Edelman R, Davis J , Sztein M, Sinigaglia F et al. CD4+ T cell clones obtained from Plasmodium falciparum sporozoite- immunized volunteers recognize polymorphic sequences of the circumsporozoite protein. J Immunol 1993; 151(1):489-499.

40. Calvo-Calle JM, Hammer J, Sinigaglia F, Clavijo P, Moya-Castro ZR, Nardin EH. Binding of malaria T cell epitopes to DR and DQ molecules in vitro correlates with immunogenicity in vivo: identification of a universal T cell epitope in the Plasmodium falciparum circumsporozoite protein. J Immunol 1997; 159(3):1362-1373.

41. Nardin EH, Calvo-Calle JM, Oliveira GA, Nussenzweig RS, Schneider M, Tiercy JM et al. A totally synthetic polyoxime malaria vaccine containing Plasmodium falciparum B cell and universal T cell epitopes elicits immune responses in volunteers of diverse HLA types. J Immunol 2001; 166(1):481-489.

42. Hunter RL, Lal AA. Copolymer adjuvants in malaria vaccine development. Am J Trop Med Hyg 1994; 50(4 Suppl):52-58.

43. Usinger WR, Lucas AH. Avidity as a determinant of the protective efficacy of human antibodies to pneumococcal capsular polysaccharides. Infect Immun 1999; 67(5):2366-2370.

44. Nardin EH, Oliveira GA, Calvo-Calle JM, Nussenzweig RS. The use of multiple antigen peptides in the analysis and induction of protective immune responses against infectious diseases. Adv Immunol 1995; 60:105-149.

45. Nardin EH, Oliveira GA, Calvo-Calle JM, Castro ZR, Nussenzweig RS, Schmeckpeper B et al. Synthetic malaria peptide vaccine elicits high levels of antibodies in vaccinees of defined HLA genotypes. J Infect Dis 2000; 182(5):1486-1496.

46. Pullen GR, Fitzgerald MG, Hosking CS. Antibody avidity determination by ELISA using thiocyanate elution. J Immunol Methods 1986; 86(1):83-87.

47. Ferreira MU, Katzin AM. The assessment of antibody affinity distribution by thiocyanate elution: a simple dose-response approach. J Immunol Methods 1995; 187(2):297-305.

48. Edelman RE, Wasserman SS, Kublin JG, Bodison SA, Nardin EH, Oliveira GA, et al. Immediate-type hypersensitivity and other clinical reactions in volunteers immunized with a synthetic multi-antigen peptide vaccine (PfCS-MAP1NYU) against Plasmodium falciparum sporozoites. Vaccine 2002; 21:269-280.

49. Birkett AJ, Lyons K, Schmidt A, Boyd D, Oliveira GA, Siddique A. Hepatitis B virus core particle containing multiple epitopes of the Plasmodium falciparum circumsporozoite protein provides a highly immunogenic malaria vaccine in preclinical analyses in rodent and primate hosts. Infect Immun 2002; 70(12):6860-6870.

50. Oliveira GA, Wetzel K, Calvo-Calle JM, Schmidt A, Birkett A, Gleiter CH, et al. Phase I trial to assess safety and immunogenicity of malariavax, a recombinant hepatitis virus core particle containing repeat and T cell epitopes Plasmodium falciparum circumsporozoite protein. Program and Abstracts of the 52nd Annual Meeting of the American Society of Tropical Medicine and Hygiene; 2003:abstract #768.

12 Appendix A: Study Events Calendar

Notes for Study Events Calendar (see Table 2 on the next page)

- B = serum β-hCG; U = urine β-hCG
- Day -28 to -1 is screening and will include history & physical examination, urinalysis, WBC with differential count, hemoglobin, platelet count, creatinine, AST, ALT, glucose, hepatitis B surface antigen, hepatitis B surface antibody, hepatitis B core antibody, hepatitis C serology, HIV serology, and serum β-hCG for female volunteers.
- Values for WBC with differential count, hemoglobin, platelet count, creatinine, AST, and ALT will be within the 7 days before first vaccination.
- Post vaccination labs include creatinine, AST, ALT, WBC with differential count, hemoglobin, and platelet count.
- Urine β-hCG will be done immediately before each vaccination for female volunteers
- Clinical exam includes, but is not necessarily limited to, taking an interim history, arm check up to day 7 post-injection, and vital signs up to day 14 post-injection.
- * implies collection of study close serology screen. This includes a repeat anti-hepatitis B surface antibody and hepatitis B surface antigen test.
- ^implies optional in-person study visit. If volunteer is present in person, blood for serology, CMI and antibody avidity will be drawn. The telephone interview will take place in-person when possible.

13 Appendix B: Volunteer Written Exam

ICC-1132 WRITTEN EXAMINATION
Volunteer Name: ____________________________________
Volunteer No.: ______________________________________

Table 2: Study Events Calendar

Study Day	Informed Consent	Vaccination	Diary	Clinical Exam	UA	CBC	Chemistry	Serology, CMI & Avidity	β-hCG	Telephone Interview
-28 to -1				X	X	X	X		X B	
0	X	X	X	X		X	X	X	X U	
1				X						
2				X		X	X			
4										X
7				X						
14						X	X	X		
28								X		
56		X	X	X		X	X	X	X U	
57				X						
58				X		X	X			
60										X
63				X						
70						X	X	X		
84				X				X		
140								X		
168		X	X	X		X	X	X	X U	
169				X						
170				X		X	X			
172										X
175				X						
182						X	X	X		
196								X		
224							*	X		
336								^		X^

I understand that the purpose of this written examination is to test my knowledge and understanding of all aspects of the study in which I am participating and to ensure the informed nature of my consent to volunteer for the study. I realize that if I score less than 70% I will not be allowed to participate as a volunteer.

Signature of volunteer: ______________________________
Date: ____/____/________
Witness: ____

Exam Score	Incorrct answers reviewed with volunteer?	Initial & Date Below
	Yes No	

1. The vaccine being tested has been approved by the Food and Drug Administration (FDA) for use in this study only.
   (a) True
   (b) False
2. The microorganism ("germ") that causes human malaria is:
   (a) A virus called influenza virus
   (b) A bacteria called Streptococcus
   (c) A fungus called Cryptococcus
   (d) A single-celled protozoan parasite called Plasmodium3)
3. Malaria is usually transmitted by:
   (a) Contaminated food
   (b) Sexual contact
   (c) Sneezing and coughing
   (d) Bite of infected mosquitoes
4. The main aim of a Phase I vaccine trial is to determine if the vaccine is safe:
   (a) True
   (b) False
5. Malaria is a public health problem because:
   (a) It causes over 200 million infections each year
   (b) It kills over 1 million people each year
   (c) Some malaria strains are becoming resistant to the medicines used to treat the infection
   (d) All of the above
6. Volunteers should not donate blood outside of the study while enrolled in the study:
   (a) True
   (b) False

7. The vaccine being tested in this trial is:
   (a) A live but weakened malaria parasite
   (b) A synthetic protein coat of the malaria parasite bound to a hepatitis B core protein
   (c) Killed whole cell malaria parasites
   (d) Malaria parasites that have been inactivated by radiation
8. The purpose of this vaccine trial is to evaluate whether this vaccine is safe and free of side effects and whether it stimulates your body to produce immunity to Plasmodium falciparum malaria.
   (a) True
   (b) False
9. This trial is designed to administer vaccine:
   (a) 3 times over 3 weeks
   (b) 3 times over 4 months
   (c) 3 times over 4 months
   (d) 3 times over 6 months
10. The vaccine will be administered:
    (a) By mouth
    (b) By injection into the thigh muscle
    (c) By injection into the muscle of the upper arm
    (d) By instilling drops into the nose
11. After vaccination volunteers should:
    (a) Report to the study nurses only if they have side effects from the vaccine
    (b) Call the study nurses daily to report temperature and come in for a blood draws 6 months and 1 year after vaccination
    (c) Come to the outpatient office for several visits to have temperature taken, give blood, and be questioned about reactions to the vaccine.
12. The most common local reactions that may occur after vaccination include:
    (a) Generalized bruising over inoculation site
    (b) Burning, redness, swelling, tenderness or subcutaneous nodules at injection site
    (c) Muscle pain, weakness, and possible loss of use of the injected arm
    (d) There are no potential local reactions to this vaccine
13. Some systemic reactions that you may experience after receiving this vaccine are:
    (a) Joint pains, jaundice, decreased red blood cell production
    (b) Fever, chills, body aches, headache, fatigue, rash, itchy hives

(c) Tingling and numbness in extremities, headache, memory loss
(d) Abdominal discomfort, vomiting, and diarrhea

14. A possible side effect of the vaccine is infection with the hepatitis B virus?
    (a) True
    (b) False

15. A possible side effect of the vaccine is testing positive for Hepatitis B core antibodies?
    (a) True
    (b) False

16. Persons who have antibodies to hepatitis B core are not allowed to donate blood in theUSA and may not be able to donate organs.
    (a) True
    (b) False

17. If I experience a reaction that might be related to the vaccine, I should:
    (a) Take 2 aspirins and call my doctor in the morning
    (b) Go to an Urgent Care Clinic or local Emergency Room
    (c) Call the study physician who can be reached 24 hours a day by pager or phone

18. Once signing the informed consent document, you cannot withdraw from the study.
    (a) True
    (b) False

19. The amount of blood drawn in this study is about the same as 2 blood bank donations.
    (a) True
    (b) False

20. Because this is a Phase 1 trial, all of the risks of this vaccine are not known at this time.
    (a) True
    (b) False

21. This experimental vaccine will eliminate the need for you to take precautions against malaria if you were to travel in a part of the world where malaria infection is common.
    (a) True
    (b) False

13.1 Answer Key to Exam

1. A
2. D
3. D
4. A
5. D
6. A
7. B
8. A
9. D
10. C
11. C
12. B
13. B
14. B
15. A
16. A
17. C
18. A
19. A
20. A
21. B

14 Appendix C: Adverse Events Grading

14.1 Abbreviations

Abbreviations utilized in the tables that follow:

ULN Upper Limit of Normal

LLN Lower Limit of Normal

Rx Medical Therapy

Req Required

Mod Moderate

IV Intravenous

ADL Activities of Daily Living

Dec Decreased

14.2 Estimating Severity Grade for Values Not in Tables, Symptom Diary or Clinical Evaluation Form

For abnormalities *not* found elsewhere in the Toxicity Tables, use the scale below to estimate grade of severity.

Grade 1 Mild; no change in activity and/or no medication necessary

Grade 2 Moderate; requires change in activity and/or medication

Grade 3 Severe; bed rest required and/or medical intervention other than medication alone (such as an outpatient visit in emergency department or clinic, excluding hospitalization)

Grade 4 Life-threatening; extreme limitation in activity, significant assistance required; significant medical intervention/therapy required, hospitalization or hospice care probable

14.3 DMID Toxicity Tables

14.3.1 Hematology

	Grade 1	Grade 2	Grade 3	Grade 4
Hgb	Hemoglobin 9.5–10.5gm/dL	8.0–9.4gm/dL	6.5–7.9 gm/dL	$<$ 6.5 gm/dL
ANC	1000–1500/mm^3	750–999/mm^3	500–749/mm^3	$<$500/mm^3
Platelets	75,000–99,999/mm^3	50,000–74,999/mm^3	20,000–49,999/mm^3	$<$20,000/mm^3
WBC	ULN–13,000/ mm^3	13,001–15,000 /mm^3	15,001–30,000/mm^3	$>$30,000 or $<$1,000 /mm^3
% PMNs	$>$ 80	90–95	$>$ 95	——

14.3.2 Chemistry and Urinalysis

	Grade 1	Grade 2	Grade 3	Grade 4
hypoglycemia	55–64 mg/dL	40–54 mg/dL	30–39 mg/dL	< 30 mg/dL or abnormal glucose *with* MS changes
hyperglycemia	> 125–160 mg/dL	161–250 mg/dL	251–500 mg/dL	> 500 mg/dl or abnormal glucose *with* DKA or seizures
hyperbilirubinemia	1.1–1.5x ULN	1.6–2.5x ULN	2.6–5x ULN	> 5x ULN
BUN	1.25–2.5x ULN	2.6–5x ULN	5.1–10x ULN	> 10x ULN
Creatinine	1.1–1.5x ULN	1.6-3.0x ULN	3.1–6x ULN	> 6x ULN or dialysis required
AST (SGOT)	> 1.5–2.5x ULN	2.6–5x ULN	5.1–10x ULN	> 10x ULN
ALT (SGPT)	> 1.5–2.5x ULN	2.6–5x ULN	5.1–10x ULN	> 10x ULN
Proteinuria	1+ or 200 mg–1 g/day	2–3+ or 1–2 g/day	4+ or 2.1-3.5 g/day	nephrotic syndrome or > 3.5 g/day
Hematuria	microscopic only, < 10 rbc/hpf	gross, no clots, > 10 rbc/hpf	gross, with clots or RBC casts	obstructive or requires transfusion

14.3.3 Cardiovascular

	Grade 1	Grade 2	Grade 3	Grade 4
Cardiac Rhythm	——	intermittent, asymptomatic, no Rx required	recurrent or persistent, symptomatic, Rx required	unstable, hospitalization & Rx required
Hypertension	transient increase > 20 mmHg; no Rx	recurrent, chronic increase > 20 mmHg, Rx required	acute Rx required, outpt Rx or hospitalization possible	end organ damage or hospitalization required
Hypotension	transient orthostatic hypotension with HR increased by < 20 bpm or decreased by < 10 mmHg SBP, No Rx	symptoms due to orthostatic hypotension or SBP decreased by < 20 mmHG, correctable with PO fluids	requires IV fluids, no hospitalization	MAP < 60 mmHg or end organ damage or shock, requires hospitalization and vasopressor Rx
Pericarditis	minimal effusion	mild/moderate asymptomatic effusion, no Rx	symptomatic effusion, pain, EKG changes	tamponade, pericardiocentesis or surgery is required
Hemorrhage	microscopic/occult	mild, no transfusion	gross blood loss, 1–2 units transfused	massive blood loss, > 3 units transfused

14.3.4 Respiratory

	Grade 1	Grade 2	Grade 3	Grade 4
Cough	transient; no Rx	persistent cough; Rx responsive	paroxysmal cough; uncontrolled by Rx	——-
Bronchospasm, acute	transient; no Rx; FEV1 70-80% of PF	requires Rx; normalizes with bronchodilator Rx; FEV1 50-70% of peak flow	no normalization with bronchodilator; FEV1 25-50% of peak flow; or retractions present	cyanosis; FEV1 < 25% of peak flow or intubation necessary
Dyspnea	dyspnea on exertion	dyspnea with normal activity	dyspnea at rest	dyspnea requiring oxygen Rx

14.3.5 Gastrointestinal

	Grade 1	Grade 2	Grade 3	Grade 4
Nausea	mild or transient; maintains reasonable intake	moderate discomfort; intake decreased significantly; some activity limited	no significant intake; requires IV fluids	hospitalization required
Vomiting	1 episode in 24 hours	2-5 episodes in 24 hours	> 6 episodes in 24 hours or needs IV fluids	physiologic consequences requiring hospitalization or requiring parenteral nutrition
Constipation	requiring stool softerner or dietary modification	requiring laxatives	obstipation requiring manual evacuation or enema	obstruction or toxic megacolon
Diarrhea	mild or transient; 3-4 loose stools/day or mild diarrhea lasting < 1 week	moderate or persistent; 5-7 loose stools/day or diarrhea lasting > 1 week	> 7 loose stools/day or bloody diarrhea or orthostatic hypotension or electrlyte imbalance or > 2L IV fluids required	hypotensive shock or physiologic consequences requiring hospitalization
Oral discomfort or dysphagia	mild discomfort; no dysphagia	some limits on eating/drinking	eating/talking very limited; unable to swallow solid foods	unable to drink fluids; requires IV fluids

14.3.6 Neurological

	Grade 1	Grade 2	Grade 3	Grade 4
Neuro-cerebellar	slight inco-ordination dysdiadochokinesis	intention tremor, dysmetria, slurred speech; nystagmus	locomotor ataxia	incapacitated
Psychiatric	mild anxiety or depression	moderate anxiety or depression; Rx required; change in normal routine	severe mood changes requiring Rx; or suicidal ideation; or aggressive ideation	acute psychosis requiring hospitalization; or suicidal gesture/attempt or hallucinations
Muscle Strength	subjective weakness no objective symptoms/ signs	mild objective signs/symptoms no decrease in function	objective weakness function limited	paralysis
Paresthesia	mild discomfort; no Rx required	moderate discomfort; non-narcotic analgesia required	severe discomfort; or narcotic analgesia required with symptomatic improvement	incapacitating; or not responsive to narcotic analgesia
Neuro-sensory	mild impairment in sensation (decreased sensation, vibratory, pinprick, hot/cold in great toes) in focal area or symmetrical distribution; or change in taste, smell, vision and/or hearing	moderate impairment (mod decreased sensation, vibratory, pinprick, hot/cold to ankles) and/or joint position or mild impairment that is not symmetrical	severe impairment (decreased or loss of sensation to knees or wrists) or loss of sensation of at least mod degree in multiple different body areas (i.e., upper and lower extremities)	sensory loss involves limbs and trunk; paralysis; or seizures

14.3.7 Musculoskeletal

	Grade 1	Grade 2	Grade 3	Grade 4
Arthralgia	mild pain not interfering with function	moderate pain, analgesics and/or pain interfering with function but not with activities of daily living	severe pain; pain and/or analgesics interfering with activities of daily living	disabling pain
Arthritis	mild pain with inflammation, erythema or joint swelling but not interfering with function	moderate pain with inflammation, erythema or joint swelling interfering with function, but not with activities of daily living	severe pain with inflammation, erythema or joint swelling and interfering with activities of daily living	permanent and/or disabling joint distruction
Myalgia	myalgia with no limitation of activity	muscle tenderness (at other than injection site) or with moderate impairment of activity	severe muscle tenderness with marked impairment of activity	frank myonecrosis

14.3.8 Skin

	Grade 1	Grade 2	Grade 3	Grade 4
Mucocutaneous	erythema; pruritus	diffuse, maculo papular rash, dry desquamation	vesiculation or moist desquamation or ulceration	exfoliative dermatitis, mucous membrane involvement or erythema, multiforme or suspected Stevens-Johnson or necrosis requiring surgery
Induration	< 15 mm	15–30 mm	> 30 mm	—–
Erythema	< 15 mm	15–30 mm	> 30 mm	—–
Edema	< 15 mm	15–30 mm	> 30 mm	—–
Rash at Injection Site	< 15 mm	15–30 mm	> 30 mm	—–
Pruritus	slight itching at injection site	moderate itching at injection extremity	itching over entire body	

14.3.9 Systemic

	Grade 1	Grade 2	Grade 3	Grade 4
Allergic Reaction	pruritus without rash	localized urticaria	generalized urticaria; angioedema	anaphylaxis
Headache	mild, no Rx required	transient, moderate; Rx required	severe; responds to narcotic Rx	requires repeated narcotic therapy
Fever	oral 37.7 - 38.5 C or 100.0 - 101.5 F	38.6 - 39.5 C or 101.6-102.9 F	39.6 - 40.5 C or103 - 105 F	> 40 C or> 105 F
Fatigue	normal activity reduced < 48 hours	normal activity decreased 25- 50% > 48 hours	normal activity decreased > 50% can't work	unable to care for self

15 Appendix D: Anaphylaxis SOP

Procedure for treatment of presenting symptoms of dyspnea, difficulty swallowing, urticaria on face and neck, severe edema, hypotension and/or weak/thready pulse due to vaccine, medication or intoxication.

- Give aqueous epinephrine 1:1000 IM 0.3 ml
- Call Code Team
- Notify MD on-call
- Vitals every five minutes or more frequently as needed
- Repeat IM epinephrine administration 0.3–0.5 ml every 5–15 minutes prn
- If respirations more difficult
  - oxygen via nasal canula @ 2 - 3 L/minute or via mask @ 5 - 8 L/minute
- Start IV normal saline

16 Appendix E: Screening Exclusion Clinical Laboratory Values

Parameter	LabCorp Reference Range	Values Leading to Study Exclusion	
		Low Outlier	High Outlier
Creatinine	0.5–1.5 mg/dl	NA	> 1.5 mg/dl
AST (SGOT)	0–40 U/L	NA	> 60 U/L (1.5x ULN)
ALT (SGPT)	0–40 U/L	NA	> 60 U/L (1.5x ULN)
Glucose	65–109 mg/dl	NA	> 125 mg/dl^
WBC	4.0–10.5 x $10^3/mm^3$	< 3.0 x $10^3/mm^3$	> 13.5 x $10^3/mm^3$
ANC	1800–7800/mm^3	< 1500/mm^3	NA
Hgb Males	12.5–17 g/dl	< 11.5 g/dl	> 20 g/dl (1.18x ULN)
Hgb Females	11.5–15 g/dl	< 10.5 g/dl	> 18 g/dl (1.18x ULN)
Hepatitis C antibody	negative	NA	positive
Hepatitis B surface antigen	negative	NA	positive
HIV antibody	negative	NA	positive
RBC casts in urine	negative	NA	positive
WBC casts in urine	negative	NA	positive
Urine protein	negative–trace	NA	≥1+

*Abnormal screening laboratory values may be repeated one time, and if normal, the repeat value will be used for screening purposes.

^3-hour fasting level defines exclusion criteria.

ULN Upper limit of normal range

NA Not applicable; "NA" means that any value is acceptable for inclusion

17 Appendix F: Volunteer Recruitment Material

17.1 Recruitment Flyers

Volunteers needed to participate in an outpatient Investigational Malaria Vaccine Study Study will require 20 outpatient visits over 9 months

You will be paid up to $1,127 for time and effort

If you are a healthy M/F 18 to 45 years old call the Center for Vaccine Development University of Maryland, Baltimore 410-706-6156

17.2 Malaria Fact Sheet

What is malaria?

Malaria is a serious, sometimes fatal, disease caused by a parasite. There are four kinds of malaria that can infect humans:

- *Plasmodium falciparum* (plaz-MO-dee-um fal-SIP-a-rum)
- *P. vivax* (VI-vacks)
- *P. ovale* (o-VOL-ley)
- *P. malariae* (ma-LER-ee-aa).

Where does malaria occur?

Malaria occurs in over 100 countries and territories. More than 40% of the people in the world are at risk. Large areas of Central and South America and the Caribbean, Africa, the Indian subcontinent, Southeast Asia, the Middle East, and Oceania are considered malaria-risk areas (an area of the world that has malaria).

How common is malaria?

The World Health Organization estimates that yearly 300–500 million cases of malaria occur and more than 1 million people die of malaria. About 1,200 cases of malaria are diagnosed in the United States each year. Most cases in the United States are in immigrants and travelers returning from malaria-risk areas, mostly from sub-Saharan Africa and the Indian subcontinent.

How do you get malaria?

Humans get malaria from the bite of a malaria-infected mosquito. When a mosquito bites an infected person, it ingests microscopic malaria parasites found in the person's blood. The malaria parasite must grow in the mosquito for a week or more before infection can be passed to another person. If, after a week, the mosquito then bites another person, the parasites go from the mosquito's mouth into the person's blood. The parasites then travel to the person's liver, enter the liver's cells, grow and multiply. During this time when the parasites are in the liver, the person has not yet felt sick. The parasites leave the liver and enter red blood cells; this may take as little as 8 days or as many as several months. Once inside the red blood cells, the parasites grow and multiply. The red blood cells burst, freeing the parasites to attack other red blood cells. Toxins from the parasite are also released into the blood, making the person feel sick. If a mosquito bites this person while the parasites are in his or her blood, it will ingest the tiny parasites. After a week or more, the mosquito can infect another person. Each year in the United States, a few cases of malaria result from blood transfusions, are passed from mother to fetus during pregnancy, or are transmitted by locally infected mosquitoes.

What are the signs and symptoms of malaria?

Symptoms of malaria include fever and flu-like illness, including shaking chills, headache, muscle aches, and tiredness. Nausea, vomiting, and diarrhea may also occur. Malaria may cause anemia and jaundice (yellow coloring of the skin and eyes) because of the loss of red blood cells. Infection with one type of malaria, *P. falciparum*, if not promptly treated, may cause kidney failure, seizures, mental confusion, coma, and death.

How soon will a person feel sick after being bitten by an infected mosquito?

For most people, symptoms begin 10 days to 4 weeks after infection, although a person may feel ill as early as 8 days or up to 1 year later. Two kinds of malaria, *P. vivax* and *P. ovale*, can relapse; some parasites can rest in the liver for several months up to 4 years after a person is bitten by an infected mosquito. When these parasites come out of hibernation and begin invading red blood cells, the person will become sick.

How is malaria diagnosed?

Malaria is diagnosed by looking for the parasites in a drop of blood. Blood will be put onto a microscope slide and stained so that the parasites will be visible under a microscope. Any traveler who becomes ill with a fever or flu-like illness while traveling and up to 1 year after returning home should immediately seek professional medical care. You should tell your health care provider that you have been traveling in a malaria-risk area.

Who is at risk for malaria?

Persons living in, and travelers to, any area of the world where malaria is transmitted may become infected.

What is the treatment for malaria?

Malaria can be cured with prescription drugs. The type of drugs and length of treatment depend on which kind of malaria is diagnosed, where the patient was infected, the age of the patient, and how severely ill the patient is at start of treatment.

How can malaria be prevented?

Visit your health care provider 4 to 6 weeks before foreign travel for any necessary vaccinations and a prescription for an antimalarial drug. Take your antimalarial drug exactly on schedule without missing doses. Prevent mosquito and other insect bites. Use DEET insect repellent on exposed skin and flying insect spray in the room where you sleep. Wear long pants and long-sleeved shirts, especially from dusk to dawn. This is the time when mosquitoes that spread malaria bite. Sleep under a mosquito bednet that has been dipped in permethrin insecticide if you are not living in screened or air-conditioned housing.

17.3 Information About Malaria Vaccine Sheet

Why develop a malaria vaccine?

Malaria is a disease caused by a parasite called *Plasmodium.* It is estimated to cause over 200 million infections and 1 million deaths each year. The infection is transmitted to people by certain types of mosquitoes. Over the past decades, these mosquitoes have become more resistant to insecticides. This has lead to mosquito population growth and an increased number of malaria infections in many parts of the world. Malaria infections can generally be treated by medicines. However, malaria parasites resistant to commonly used medicines are increasingly found. Because of these facts, vaccines to prevent malaria infection are being developed.

What are the purposes of this study?

This study will evaluate the safety of and immune responses to an investigational malaria vaccine called ICC-1132.

What is ICC-1132?

It is an experimental malaria vaccine produced in bacteria called *E. coli.* The vaccine consists of a protein to which malaria antigens (substances that stimulate production of antibodies) have been attached. The protein is an internal component of the hepatitis B virus. The malaria antigens are derived from the protein coat of the malaria parasite. Binding the hepatitis protein to the malaria protein increases antibodies against malaria in animals.

What does the study entail?

Forty-eight volunteers will be immunized with the vaccine. Thirty-three (33) volunteers will be assigned to receive ICC-1132 + aluminum hydroxide (alhydrogel) adjuvant. Eight (8) volunteers will receive the 20 μg dose and 25 volunteers with receive the 50 μg dose. An injection of the assigned vaccine dose will be given to each volunteer on days 0, 56 and 168 (a total of 3 injections). Volunteers receiving the 50 μg dose of vaccine will be invited to participate in a subsequent malaria challenge study. Your participation in any subsequent study is not mandatory and is in no way required for participation in this present study. For 7 days after each vaccination you will complete a symptom diary each evening to report any symptoms that you experience. You will return to the clinic for a check up on days 1, 2, 7 and 14 after each injection. You will have a total of 15–17 blood draws over 8 months. The total amount of blood drawn will be approximately 860 cc—about as much as 2 blood bank donations. You will be reimbursed for your time and effort spent to participate in the study. This will amount to $1,127 for successful completion of all study visits.

Who can participate in the study?

To qualify for this study, you must be 18 to 45 years of age and in good general health as determined by history, physical examination, blood tests, and urine tests. Pregnant or breastfeeding women are not eligible to participate.

Are there are risks?

As with any vaccine, redness, swelling, tenderness and, rarely, infection may occur at the site of injection. Tenderness, bruising, or fainting may result from blood draws. If the vaccine is given subcutaneously (under the skin), an inflammatory nodule can form. Because of this possibility, injections will be given into the deltoid muscle of the arm. Allergic reactions are possible. Anaphylaxis, a severe allergic reaction that is potentially serious and may even be fatal, occurs rarely. The vaccine may induce antibodies to hepatitis B

core antigen. This may make you ineligible for blood donation and may lead to confusion if you are ever evaluated as a potential organ donor.

Why participate?

Your participation may contribute to the development of a safe and effective vaccine against malaria.

17.4 Overview of Each Study Visit and Estimated Time Required

See Table 3 on the following page

18 Appendix G: Continued Vaccination

18.0.1 Individual

INVESTIGATOR CHECKLIST FOR CONTINUED VACCINATION (INDIVIDUAL)

Mark whether any of the following occurred after the last vaccination

Yes/No Anaphylaxis or bronchospasm requiring medical therapy within 72 hours of inoculation.

Yes/No Any systemic rash, including but not limited to urticaria, generalized petechiae, or erythema multiforme, not attributable to another cause.

Yes/No Any Serious Adverse Event, Probably or Definitely related to vaccination.

Yes/No Local reaction at the injection site (pain or pruritus) Grade 3, for more than one consecutive day.

Yes/No Any symptom rated at Grade 3 or higher, Possibly, Probably or Definitely related to vaccination.

Yes/No Any objective physical finding or laboratory abnormality of Grade 3 or higher, Possibly, Probably or Definitely related to vaccination.

Comments: If any of the items are marked “Yes”, suspend further immunization of the individual pending review of safety data.

Report all “Yes” reactions to the Safety Monitoring Committee and NIAID within 5 days, any SAE within 24 hours.

Reported to Safety Monitoring Committee: ____ / ____ / ____ (mm/dd/yyyy) _____Initials

Reported to NIAID: ____ / ____ / ____ (mm/dd/yyyy) ______Initials

YES/NO REVIEWED AND REACTIONS ACCEPTABLE FOR CONTINUED VACCINATION OF THE INDIVIDUAL?

Signature of Investigator:

____/____/____ (mm/dd/yyyy)

Table 3: Visit Overview

Visit Number	Study Day	Days after last vaccine	Primary Visit Aim	Blood or Urine Test Done?	Physical Exam or Arm Check Done?	Estimated Visit Time
1	-28 to -1	NA	Health Screening & Informed Consent	Yes	Yes	2 hours
2	0	NA	Vaccination Number 1	Yes	Yes	1.5 hours
3	1	1	Clinical Follow-up	No	Yes	20 minutes
4	2	2	Clinical Follow-up	Yes	Yes	30 minutes
5	4	4	Telephone Follow-up	No	No	15 minutes
6	7	7	Clinical Follow-up	No	Yes	20 minutes
7	14	14	Clinical Follow-up	Yes	Yes	30 minutes
8	28	28	History & Blood Draw	Yes	No	20 minutes
9	56	0	Vaccination Number 2	Yes	Yes	1.25 hours
10	57	1	Clinical Follow-up	No	Yes	20 minutes
11	58	2	Clinical Follow-up	Yes	Yes	30 minutes
12	60	4	Telephone Follow-up	No	No	15 minutes
13	63	7	Clinical Follow-up	No	Yes	20 minutes
14	70	14	Clinical Follow-up	Yes	Yes	30 minutes
15	84	28	History & Blood Draw	Yes	No	20 minutes
16	140	84	History & Blood Draw	Yes	No	20 minutes
17	168	0	Vaccination Number 3	Yes	Yes	1.25 hours
18	169	1	Clinical Follow-up	No	Yes	20 minutes
19	170	2	Clinical Follow-up	Yes	Yes	30 minutes
20	172	4	Telephone Follow-up	No	No	15 minutes
21	175	7	Clinical Follow-up	No	Yes	20 minutes
22	182	14	Clinical Follow-up	Yes	Yes	30 minutes
23	196	28	History & Blood Draw	Yes	No	20 minutes
24	224	56	History & Blood Draw	Yes	No	20 minutes
25	336	168	Telephone Follow-up	No	No	15 minutes

18.0.2 Cohort

INVESTIGATOR CHECKLIST FOR CONTINUED VACCINATION (COHORT)

Mark whether any of the following occurred after the last vaccination

Yes/No Anaphylaxis or bronchospasm requiring medical therapy within 72 hours of inoculation in *any* volunteer.

Yes/No Any systemic rash, including but not limited to urticaria, generalized petechiae, or erythema multiforme, not attributable to another cause in *any* volunteer.

Yes/No Any Serious Adverse Event, Probably or Definitely related to vaccination in *any* volunteer.

Yes/No *Three or more subjects in a single dose cohort* reported a symptom rated at Grade 3 or higher, Possibly, Probably or Definitely related to vaccination.

Yes/No *Two or more subjects in a single dose cohort* experienced an objective physical finding or laboratory abnormality of Grade 3 or higher, Possibly, Probably or Definitely related to vaccination.

Comments: If any of the items are marked "Yes", suspend further immunization of the individual pending review of safety data.

Report all "Yes" reactions to the Safety Monitoring Committee and NIAID within 5 days, any SAE within 24 hours.

Reported to Safety Monitoring Committee: ____ / ____ / ____ (mm/dd/yyyy) _____Initials

Reported to NIAID: ____ / ____ / ____ (mm/dd/yyyy) ______Initials

YES/NO REVIEWED AND REACTIONS ACCEPTABLE FOR CONTINUED VACCINATION OF THE COHORT?

Signature of Investigator:

____/____/____ (mm/dd/yyyy)
